# Brush-like Polymer Prodrug with Aggregation-Induced Emission Features for Precise Intracellular Drug Tracking

**DOI:** 10.3390/bios12060373

**Published:** 2022-05-29

**Authors:** Sanaz Naghibi, Soheila Sabouri, Yuning Hong, Zhongfan Jia, Youhong Tang

**Affiliations:** 1 Institute for NanoScale Science and Technology, College of Science and Engineering, Flinders University, Tonsley, SA 5042, Australia; sanaz.naghibi@flinders.edu.au; 2 Department of Biochemistry and Chemistry, La Trobe Institute for Molecular Science, La Trobe University, Bundoora, VIC 3086, Australia; s.sabouri@latrobe.edu.au (S.S.); y.hong@latrobe.edu.au (Y.H.); 3 Australia-China Joint Research Centre on Personal Health Technologies, Tonsley, SA 5042, Australia

**Keywords:** brush-like polymer, aggregation-induced emission, prodrug, cell imaging

## Abstract

In this study, a brush-like polymer with aggregation-induced emission (AIE) features was synthesized for drug delivery and intracellular drug tracking. The polymer consisting of tetraphenylethene (TPE) chain-end as well as oligo-poly (ethylene glycol) (PEG) and hydrazine functionalities was successfully synthesized through copper (0)-mediated reversible-deactivation radical polymerization (Cu^0^-mediated RDRP). Anticancer drug doxorubicin (DOX) was conjugated to the polymer and formed a prodrug named TPE-PEGA-Hyd-DOX, which contains 11% DOX. The hydrazone between DOX and polymer backbone is a pH-sensitive linkage that can control the release of DOX in slightly acidic conditions, which can precisely control the DOX release rate. The drug release of 10% after 96 h in normal cell environments compared with about 40% after 24 h in cancer cell environments confirmed the influence of the hydrazone bond. The ratiometric design of fluorescent intensities with peaks at 410 nm (emission due to AIE feature of TPE) and 600 nm (emission due to ACQ feature of DOX) provides an excellent opportunity for this product as a precise intracellular drug tracker. Cancer cells confocal microscopy showed negligible DOX solution uptake, but an intense green emission originated from prodrug uptake. Moreover, a severe red emission in the DOX channel confirmed a promising level of drug release from the prodrug in the cytoplasm. The merged images of cancer cells confirmed the high performance of the TPE-PEGA-Hyd-DOX compound in the viewpoints of cellular uptake and drug release. This polymer prodrug successfully demonstrates low cytotoxicity in healthy cells and high performance in killing cancer cells.

## 1. Introduction

Cancer has become the leading cause of death worldwide as well as a major health concern facing mankind. Despite several modern cancer therapy techniques, chemotherapy remains the preferable and most reliable method to tackle some types of cancer, showing the effective capability of preventing the rapid development of cancer cells. However, cancer survivors might experience some side effects and severe consequences [1,2]. Drug delivery systems (DDSs) have been developed to eliminate, or at least reduce, the side effects of cancer therapy procedures by the controlled release of theranostic agents in target tumors or tissues [3]. In general, DDSs are biocompatible and capable of the cellular uptake and release of anticancer drugs under specific circumstances. Metal-organic frameworks (MOFs), metal oxide nanoparticles, liposomes, and hydrogels are some examples of carriers that have been reported in different DDSs [4,5,6,7,8]. Other types of carriers, mainly organic compounds, are designed by conjugating a polymer or amphiphilic block copolymer with the intended theranostic agents. Molecular engineering and sophisticated chemical modification methods are prerequisites for the preparation of these compounds with high efficiency and precision in drug delivery applications. Among diverse types of polymers, polyethylene glycol (PEG) is the first FDA-approved polymer for drug delivery due to its high-water solubility, low immunogenicity, and antigenicity [9,10,11,12]. Although PEG has several advantages and shows high performance in drug loading, its linear structure in comparison with brush-like polymers is a concern and limits its loading capacity due to the existence of only two ends in each PEG chain [13]. Typically, a linear polymer molecule has two free ends. Tethering one of these ends to another polymer molecule leads to the formation of a macromolecular structure with more than two free ends. As more polymer chains are linked to the backbone polymer, more free ends will be available. Polymer brushes with different structures and morphologies demonstrate interesting surface-related behaviors, which have led to the introduction of some new functionalities that are mostly related to the number of repeating units [14]. An anticancer drug, known as DOX-PEG-alendronate with a core-shell structure, has been reported by Ye et al. Due to the hydrophobic nature of doxorubicin (DOX) [15], this prodrug forms micelles with a low loading capability of other features and functions. To afford the drug delivery system with multiple functions, polymer prodrugs were linked to photocatalyst nanoparticles to generate reactive oxygen species (ROS) in photodynamic therapy [16] or equipped with receptor-targeting agents for enhanced cancer therapy [17].

Aggregation-induced emission (AIE) is a novel photophysical phenomenon coined in 2001, which is non-emissive in the solution state but becomes highly emissive upon aggregation. In contrast to the usual fluorophores notion of aggregation-caused quenching (ACQ), the fluorescent emission of an organic fluorogen would be quenched in solid-state or aggregated form [18,19]. Strong emission, even at very low concentrations in the solid-state, demonstrates powerful and real-time monitoring potential in both in vitro and in vivo systems [20,21,22]. Due to the trend of utilizing various types of polymers in vitro trials or even in approved medical procedures, early studies have been implemented to monitor different aspects of AIE polymers in biosystems [23]. In 2013, one of the first reports was published by Chen et al. Here, the authors designed a fluorescent micelle, including block copolymers, poly (ethylene glycol)-b-poly[styrene-co-(2-(1,2,3,4,5-pentaphenyl-1H-silol-1-yloxy) ethyl methacrylate) and DOX or PEG-b-P(S-co-PPSEMA)-DOX. The DOX loading capacity of the synthesized carrier and encapsulation efficiency were monitored by recording the emission of the prodrug [24]. In 2014, Zhang et al. designed traceable nano-formulations based on tetraphenylethene (TPE) as an AIE agent, with the DOX release efficiency of about 15% [25]. In 2014, Yuan et al. reported a prodrug composed of cisplatin (Pt(ii)), DOX, cyclic arginine-glycine-aspartic acid tripeptide (cRGD), and TPE, in which cisplatin (Pt(ii)) and DOX were utilized as anticancer drugs, cRGD as a targeting ligand and TPE as a tracking agent. This prodrug showed a clear trace in confocal microscopy images and its DOX releasing ratio in the presence of ascorbic acid was about 80% [26]. Moreover, different AIEgens were utilized, such as TPE [27,28,29], hyperbranched polyamide amine (H-PAMAM) [30], tricyano-methylene-pyridine (TCM) [31], triphenylamine (TPA) [32], fluorene-TPE (FLU-TPE or FTP) [33], etc. In most of these works, due to the use of a linear polymer, the synthesized macromolecules possess a limited number of chain-ends, which is a key factor in the determination of the loading capacity of a carrier.

In this research, we aimed at treating this deficiency and trying to improve the loading capacity of the drug carrier using a brush-like polymer with multifunctional units as a backbone of the targeted compound. Due to the existence of several side chains and the capability of holding different components, loading the AIE moiety and anticancer drug would be manageable. Taking advantage of tetraphenylethene bromoisobutyrate (TPEBIB, an AIEgen with blue emission) and DOX (an ACQ fluorescence compound with red emission), [34] could lead to the development of a DDS that complies with the ratiometric emission protocol and provides the real-time monitoring possibility of targeting, drug-release, cellular uptake, and cancer cell viability. To achieve this aim, a new brush-like carrier with the AIE feature has been designed and prepared. TPEBIB was synthesized and used as an initiator for copolymerization of poly (ethylene glycol) acrylate (PEGA) and hydrazine monomers through the copper (0)-mediated reversible-deactivation radical polymerization (Cu^0^-mediated RDRP). Then, DOX was conjugated to the carrier through hydrazone bonds. The synthesized compound and its drug loading capacity, drug release profile, cytotoxicity and cellular uptake were investigated. Finally, a brief summary is provided to demonstrate the real-time monitoring capability of the synthesized compound for an in vitro application.

## 2. Materials and Methods

### 2.1. Preparation Methods

All of the materials were purchased from Sigma-Aldrich (Castle Hill, NSW, Australia) unless otherwise stated: Benzophenone, 4-hydroxybenzophenone (ChemSupply, Gillman, SA, Australia), zinc powder, tetrahydrofuran (THF), TiCl_4_ (ChemSupply, Gillman, SA, Australia), dichloromethane, triethylamine (TEA), 2-bromoisobutyryl bromide (BIBB), acrylic acid (AA), tert-butyl carbazate (TBC), N-(3-dimethylaminopropyl)-N’-ethylcarbodiimide hydrochloride (EDC), copper bromide (CuBr_2_), Tris [2-(dimethylamino)ethyl] amine (Me_6_TREN), dimethyl sulfoxide (DMSO), etc. All of the chemicals were used as received, except for THF and poly (ethylene glycol) methyl ether acrylate (PEGA, average Mn 480). Here, THF was dried over calcium hydride powder and PEGA was passed through an alumina column to remove its monomethyl ether hydroquinone inhibitor (MEHQ). The methodology used in this research includes the preparation of an initiator with the AIE trait, hydrazine monomer synthesis, performing polymerization of monomers, forming the targeted AIE-polymer, and loading the anticancer drug.

**Initiator preparation:** Tetraphenylethene bromoisobutyrate (TPEBIB) is an AIEgen [35], which also plays the role of an initiator. To synthesize this compound, TPEOH was prepared in accordance with the literature [36]. Briefly, benzophenone, 4-hydroxybenzophenone (ChemSupply, Gillman, SA, Australia) and zinc powder were dissolved in THF. Then, with extreme precautions, TiCl_4_ (ChemSupply, Gillman, SA, Australia) was injected gradually at ~−80 °C within 1 h. The mole ratio of benzophenone:4-hydroxybenzophenone:zinc powder:TiCl_4_:THF was 11:11:30:13:1500. Thereafter, the as-achieved suspension was slowly heated to ~25 °C and quenched using dilute HCl. Following this step, extraction using dichloromethane, washing with brine and purification by a silica-gel column and hexane/ethyl acetate eluent (3/1) was accomplished to achieve a crud compound of TPEOH. The synthesized compound was characterized by ^1^H-NMR, (Figure S1, see the Supplementary Materials), 7.09–7.05 ppm (m, 9H), 7.09 ppm (m, 6H), 6.96–6.93 ppm (d, 2H) and 6.60 ppm (d, 2H).

To take advantage of this compound in the targeted drug carrier, a modification was required to convert it into an initiator. As Br-containing initiators are more effective in the RDRP process, TPEBIB was synthesized in accordance with the literature [35]. Briefly, 5 g of TPEOH and TEA were added to THF at the temperature of 0 °C in an ice bath. Then, a solution of BIBB was added drop wisely. The mole ratio of TPEOH:TEA:BIBB:THF was 35:42:52:1500. Thereafter, the suspension was stirred at 0 °C for 30 min and then it remained at room temperature for 48 h. Following this step, the mixture was filtered and washed with diethyl ether. Rotary evaporation of the mixture resulted in a yellow liquid, which was further purified by column chromatography using hexane/ethyl acetate (3/1) as eluent. ^1^H-NMR was utilized for assessing the TPEBIB formation (Figure S2), 7.14–7.09 (m, 9H), 7.07–6.93 (m, 6H), 6.92 (d, 2H) and 2.08 (s, 1H). Figure S3 shows the molecular structures and the process used for synthesizing TPEBIB from benzophenone, 4-hydroxybenzophenone, and BIBB.

**Tert-butyl-2-acryloylhydrazine-1-carboxylatesynthesis as a hydrazine monomer:** The hydrazone bond has been widely utilized for prodrug designing, due to its capability to attach to DOX molecules, form a reversible bond and its high sensitivity to pH variation [37,38]. To add this feature to the targeted compound, a hydrazide derivative was synthesized and used as a monomer in the polymerization process [39]. Briefly, acrylic acid (AA) and tert-butyl carbazate (TBC) were dissolved in an H_2_O-THF mixture. N-(3-dimethylaminopropyl)-N’-ethylcarbodiimide hydrochloride (EDC) was added gradually and remained under stirring for 3 h. The mole ratio of AA:TBC:EDC:THF:H2O was 82:99:92: 1108:10,000. The crude product was extracted three times with ethyl acetate and then the organic layer was washed three times with 0.1 M HCl, once with H_2_O and two times with brine. The as-achieved organic phase was dried using anhydrous Na_2_SO_4_ and the solvent was removed under vacuum. The residual solid product was purified by the recrystallization process using ethyl acetate from 70 to 25 °C. A white crystalline powder was afforded (denoted as tert-butyl-2-acryloylhydrazine-1-carboxylate). ^1^H-NMR and ^13^C-NMR were utilized to evaluate the synthesized compound. ^1^H-NMR (Figure S4) 8.25 (s, 1H), 6.22–6.93 (m, 2H), 5.78 (dd, 1H) and 1.52 (s, 9H), as well as ^13^C-NMR (Figure S5) 164.8 (s), 155.7 (s), 128.6 (d), 127.8 (t), 82.1 (s) and 28.2 (q) confirmed the formation of the targeted tert-butyl-2-acryloylhydrazine-1-carboxylate compound. Figure S6 shows the molecular structures and the process used for synthesizing hydrazine monomers using AA and TBC.

**Copolymer synthesis using the Cu^0^-mediated RDRP:** PEGA was selected as a monomer to form a brush-like structure during its polymerization [40]. Briefly, 0.15 equivalents copper bromide (CuBr_2_) and 0.9 equivalents Tris [2-(dimethylamino) ethyl] amine (Me_6_TREN) were added to 0.2 mL dimethyl sulfoxide (DMSO) and agitated completely. Then, 40 equivalents PEGA and 1 equivalent TPEBIB were dissolved in 1.35 mL acetone. The CuBr_2_-Me_6_TREN and PEGA-TPEBIB solutions were mixed and purged with N_2_ for 15 min. Then, 50 mm of a pre-activated copper wire (Cu^0^) was wrapped around a magnet bar and added to the stirring solution. After 50 min, 10 equivalents hydrazine monomer, 0.5 mL DMSO and 0.2 mL acetone were mixed and added to the stirring solution. After 3.5 h of stirring, the obtained compound passed through a short neutral alumina column to remove the dissolved copper salts, then precipitated using diethyl ether and hexane. The achieved product of this polymerization process was evaluated by FTIR and GPC.

**Drug loading:** This is the final step in the preparation of prodrug. Here, deprotection of the synthesized compound is required before DOX loading. The hydrazone functional groups are sites where DOX conjugation could be performed. Although the synthesized compound contains the hydrazine motif, it should be functionalized by the tert-butyl group deprotection [39]. To achieve this aim, 0.2 g of the synthesized copolymer was dissolved in 2 mL dichloromethane (DCM) and then 2 mL trifluoroacetic acid (TFA) was added gradually to the solution. The solution color changed to yellow after 2 h of stirring at room temperature. N_2_ gas stream was utilized to remove TFA, resulting in an oily liquid. This was diluted in 3 mL of water to achieve a solid phase. The neutralization of the synthesized salt was accomplished by NaHCO_3_ and changed into a colorless solution. Thereafter, 2 h of stirring was applied to determine that all of the chemical reactions have occurred. Then, the obtained compound was dialyzed against water using SnakeSkin™ dialysis tubing (3500 MWCO, 22 mm, Thermo Scientific, WA, Australia). Finally, the sample was dried by lyophilization.

The previous procedure led to the achievement of the targeted drug carrier and the obtained sample was considered for the uptake of Adriamycin (CAS: 25316-40-9, AdooQ Bioscience, Irvine, California, USA). For this purpose, 0.12 g of the synthesized polymer sample was dispersed in 2 mL methanol. Then, two drops of acetic acid glacial and 34 mg DOX were added into the stirring mixture for 48 h under stirring at 50 °C, followed by dialyzing against deionized water for 2 days (MWCO 3500). The obtained red color product was collected and dried by lyophilization and stored in the fridge at about 4 °C. FTIR and ^1^H-NMR were the techniques utilized for the evaluation of the deprotection procedure or drug loading process.

### 2.2. Characterization

^1^H-NMR and ^13^C-NMR spectra were collected using a Bruker 600 MHz spectrometer. FTIR spectra was performed on a Thermo Nicolet NEXUS 870 ESP FT-IR/FT-NIR spectrometer. GPC results were achieved from a Waters Alliance e2695 separations module, PL spectra were measured by an Agilent Cary Eclipse fluorescence spectrometer, and DLS was conducted by a Malvern Panalytical zeta sizer.

Drug release assay was accomplished based on the measurement of the absorbance of DOX solution using a UV–Vis spectrometer (Agilent Cary 60 UV–Vis Spectrophotometer). To achieve this aim, an adequate amount of the synthesized compound was dissolved in water to reach the concentration of 5 g/L. Then, two dialysis bags (MWCO 3500) were filled with the solution, 1 mL in each, and placed in two beakers containing 15 mL phosphate-buffered saline (PBS) with pH values of 7.4 and 5.9. The temperature of the solution should remain at constant temperature at 37 °C. The absorbance values of the buffer solutions after different times were considered as an index that shows the drug release performance. The calibration method was performed by some pristine DOX solutions with distinct concentrations at 2, 5, 10, 20, 30, 40 and 50 mg/L. Figure S7 shows the calibration curve and a mathematical relation between the observed absorbance and DOX concentration, with y = 53.624x − 0.6212, where x and y are the absorbance values and DOX concentration (mg/L), respectively. At first, the synthesized sample was dissolved in water to reach a concentration of 500 mg/L. Then, the obtained solution was subjected to UV–Vis spectrometer and the calibration equation was used to estimate the DOX concentration. Therefore, a DOX concentration of 55.17 mg/L was obtained. This indicates that the weight ratio of DOX in the synthesized compound is 11%. In accordance with this value, the drug release percentage could be measured and reported. Here, 1 mL of a solution with a concentration of 5 g/L contains 550 μg DOX. If 100% of DOX is released, passed through the dialysis membrane, and dissolved in the buffer solution (15 mL), the resulting concentration will be 37 mg/L. In accordance with the calibration equation, the corresponding absorbance for this concentration would be 0.702. Therefore, the DOX release percentage during drug release would be achieved by Equation (1).

DOX release % = (absorbance at λ_max_/37) × 100 (1)

For cell viability assay, 4 × 10^4^ cells were seeded into a 96-well tissue culture plate in the presence of 100 μL DMEM supplemented with 10% FBS at 37 °C and 5% CO_2_ overnight. Then, the prodrug, TPE-PEGA-Hyd polymer, and pristine DOX solution were added separately in various concentrations (0, 0.0001, 0.001, 0.01, 0.1, 1, 10 and 100 μg/mL) and remained at constant temperature for 24, 48 and 72 h. Cell viability assessment was conducted using Trypan Blue (Thermo Fisher Scientific, WA, Australia). Each data point was recorded from 12 replicates of a single microplate. The effects of the incubation time and the mentioned concentration on the percentage of cytotoxic cells of 12 independent samples were reported based on the mean of cell viability values. The corresponding standard deviation values for data points were presented in Table S1.

For cell staining and imaging, TPE-PEGA-Hyd-DOX prodrug or TPE-PEGA-Hyd polymer were dissolved in PBS (1 g/L), whereas DOX was dissolved in DMSO (5 mM). Then, the solutions remained at constant temperature at −20 °C in the dark. Thereafter, 6 × 10^4^ cells were plated on an ibidi μ-slide 8-well plate for fixed cells imaging. After 24 h, the cells were ready to be applied to the treatments. HeLa cells (6 h) and NIH3T3 cells (24 h) were treated with the freshly diluted prodrug, TPE-PEGA-Hyd polymer and pristine DOX solution (10 μg/mL) at 37 °C and 5% CO_2_. After each treatment, cells were washed with PBS before they were fixed with 4% (*v*/*w*) paraformaldehyde (PFA) in PBS for 15 min at room temperature. Imaging was conducted by a confocal laser-scanning microscope (LSM 800, Zeiss, Oberkochen, Germany) using a 63× objective lens. In the case of confocal microscopy, the excitation wavelengths for DOX and TPE channels were 488 and 405 nm, whereas their emission wavelength ranges were 570–620 and 450–520 nm, respectively.

## 3. Results and Discussion

### 3.1. Synthesis of TPE-PEGA-Hydrazine Polymer Compound

**Figure 1:**
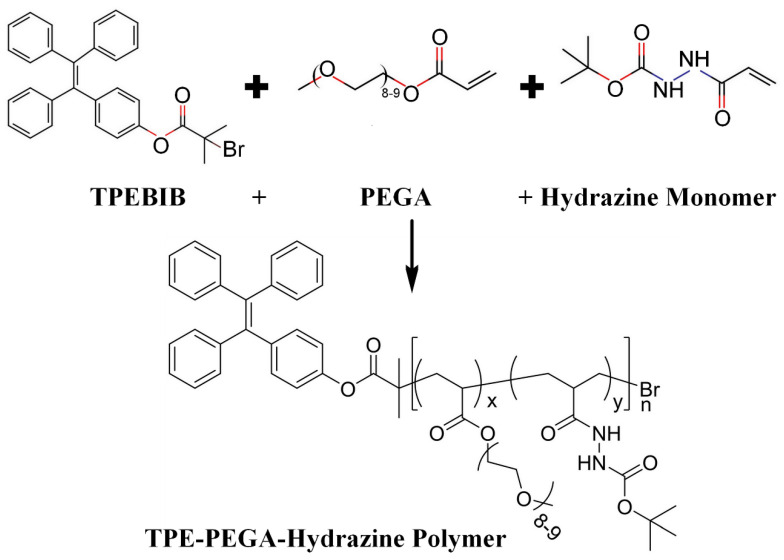
Synthesis pathway of AIE functional polymer, TPE-PEGA-Hyd.

**Figure 2:**
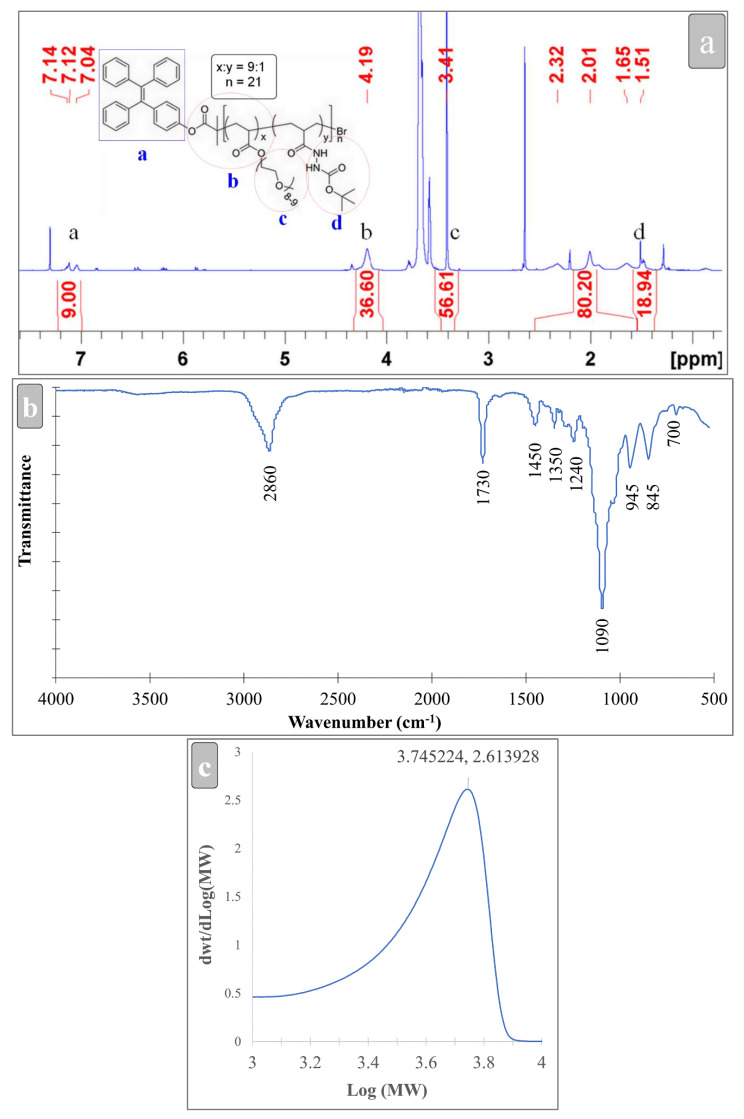
Characterization of the synthesized TPE-PEGA-Hyd polymer. (**a**) ^1^H-NMR, (**b**) FTIR and (**c**) GPC spectra.

TPEBIB, as a functional initiator in the polymerization reactions, provided the necessary conditions to polymerize the PEGA and hydrazine monomers. Figure 1 shows the molecular structure and sequence of the copolymerization process. In accordance with the TPE-PEGA-hydrazine structure, the degree of polymerization or ‘*n*’ denotes the number of side chains linked together in a brush-like structure. Moreover, ‘x’ and ‘y’ show the free PEGA end situations and free hydrazine end situations. Figure 2a–c illustrates the ^1^H-NMR, FTIR and GPC spectra of the TPE-PEGA-Hyd polymer, respectively. In Figure 2a, an evaluation of the ^1^H-NMR results shows that the polymerization degree or n is 21 and the x:y ratio is 9.5. This indicates that 19 units of PEGA side chain end and 2 units of hydrazine end exist in each polymer chain. Figure S8 shows a schematic of this brush-like structure. This achievement confirms the high capacity of the synthesized compound for loading different types of functionalities. In accordance with the n value, the molecular weight of the synthesized compound was estimated as 10,000 g·mol^−1^ (Mn,_NMR_ = 10^4^ g·mol^−1^), whereas the GPC measurement provided an estimation of 5500 g·mol^−1^ ($\bar{\mathrm{Mw}}$,_GPC_ = 5500 g·mol^−1^). The lower molecular weight from GPC is due to the brush-liked chain structure of our polymer and the use of linear polystyrene as a standard.

In Figure 2b, the FTIR spectrum shows the existence of transmittance at 2860, 1730, 1450, 1350, 1240, 1090, 945 and 845 cm^−1^. A relatively wide band at 2860 cm^−1^ is related to the aromatic C-H stretching vibration of the PEGA main chain, a band at 1730 cm^−1^ is related to the C=O stretching vibration of ester moieties, a strong band at 1090 cm^−1^ is related to the C-O stretching vibration of PEG and finally a weak band at 845 cm^−1^ is related to the C=C bending vibration of the ester bond. These four bands mainly originated from PEGA [41]. Two bands at 1350 and 1240 cm^−1^ are related to the symmetric and asymmetric C-N stretching vibration of the hydrazine component [42]. A slightly weak band at 1450 cm^−1^ might be related to the aromatic C-H stretching vibration and a weak band at 945 cm^−1^ could be related to the C=C bending vibration. These bands mainly originated from TPEBIB [35].

### 3.2. Drug Loading

**Figure 3:**
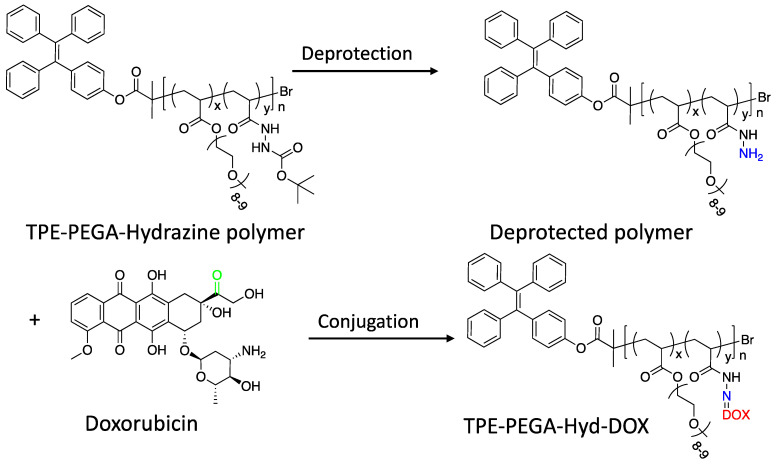
Deprotection of the synthesized polymer, followed by the DOX loading process to achieve the TPE-PEGA-Hyd-DOX prodrug. The yellow highlighted ends show the conjugation spot of deprotected compound and DOX to form the prodrug.

**Figure 4:**
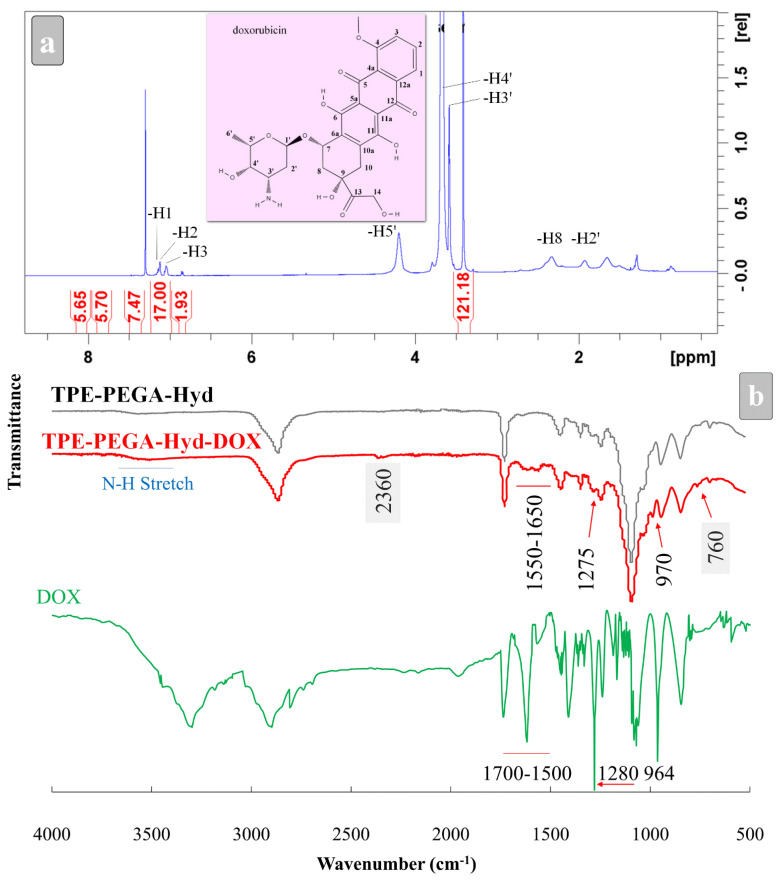
Characterization of the prodrug. (**a**) ^1^H-NMR and (**b**) FTIR spectra. The molecular structure of DOX and FTIR spectra of the synthesized polymer and DOX were provided for comparison.

In this step, an anticancer drug should be attached to the hydrazine end of the TPE-PEGA-Hyd polymer. To achieve this aim, an activation process is required, namely deprotection. As illustrated in Figure 3, DOX could be loaded on the deprotected hydrazine moiety to form the TPE-PEGA-Hyd-DOX compound. The brush-like structure of the synthesized compound could be observed in Figure 3, showing its high performance to load DOX molecules. Figure 4a,b shows the ^1^H-NMR and FTIR spectra of the TPE-PEGA-Hyd-DOX compound, respectively. A comparison between Figure 2a and Figure 4a shows the effect of deprotection and drug loading steps on the ^1^H-NMR spectra of the synthesized TPE-PEGA-Hyd and TPE-PEGA-Hyd-DOX compounds. Two events occurred during these actions. First, one chemical shift at 1.51 ppm was observed in the polymer spectrum related to the hydrazine part, whereas there is no trace in the drug-loaded compound. Moreover, drug loading led to the intensification of the chemical shifts at 7.04, 7.12 and 7.14 ppm. The former might be related to the deprotection process, which leads to the removal of the tert-butyl moiety, while the latter originated from the existence of H in positions 3, 2 and 1 in DOX structure, as shown in Figure 4a.

Figure 4b shows the FTIR spectra of the TPE-PEGA-Hyd polymer prior to the deprotection (grey color), as well as after DOX loading (red color) and pristine DOX (black color). Although FTIR spectra before and after drug loading are almost the same, there are some important minor differences, especially in the 1700–500 cm^−1^ range, which show the existence of DOX in the synthesized compound. Peaks observed at 970, 1275 and 1550–1650 cm^−1^ range in the TPE-PEGA-Hyd-DOX compound are in good agreement with the corresponding peaks of the pure DOX. Moreover, the intensification of the shoulder located at around 3500 cm^−1^, which is related to the N−H stretching bond, could be considered as evidence of conjugation between the deprotected hydrazide group in the TPE-PEGA-Hyd polymer and DOX, as highlighted in Figure 3. Of note, the peaks at 760 and 2360 cm^−1^ are not important for consideration, since the first peak is in the fingerprint region and the second is related to CO_2_. From the above discussion, the issues are intended to confirm the formation of the TPE-PEGA-Hyd-DOX compound. Subsequently, the synthesized compound would be characterized as an anticancer smart drug with AIE trait.

Of note, the DOX loading amount in this work (i.e., 11%) is less than those reported by other research groups. For instance, a compound containing 20% DOX has been reported by Chen et al. [24]. This difference is due to the different methods that have been utilized for drug loading. Mixing DOX-HCl and triethylamine for conversion into a free base and then loading the mixture as a shell on a hydrophobic core is a high-performance method that has been used by the abovementioned research group. On the other hand, in this research, the hydrazone bond has been used as a tool for controlling drug loading and release. This bond between the carrier and DOX plays a key role in controlling drug release kinetics.

### 3.3. Drug Release

**Figure 5:**
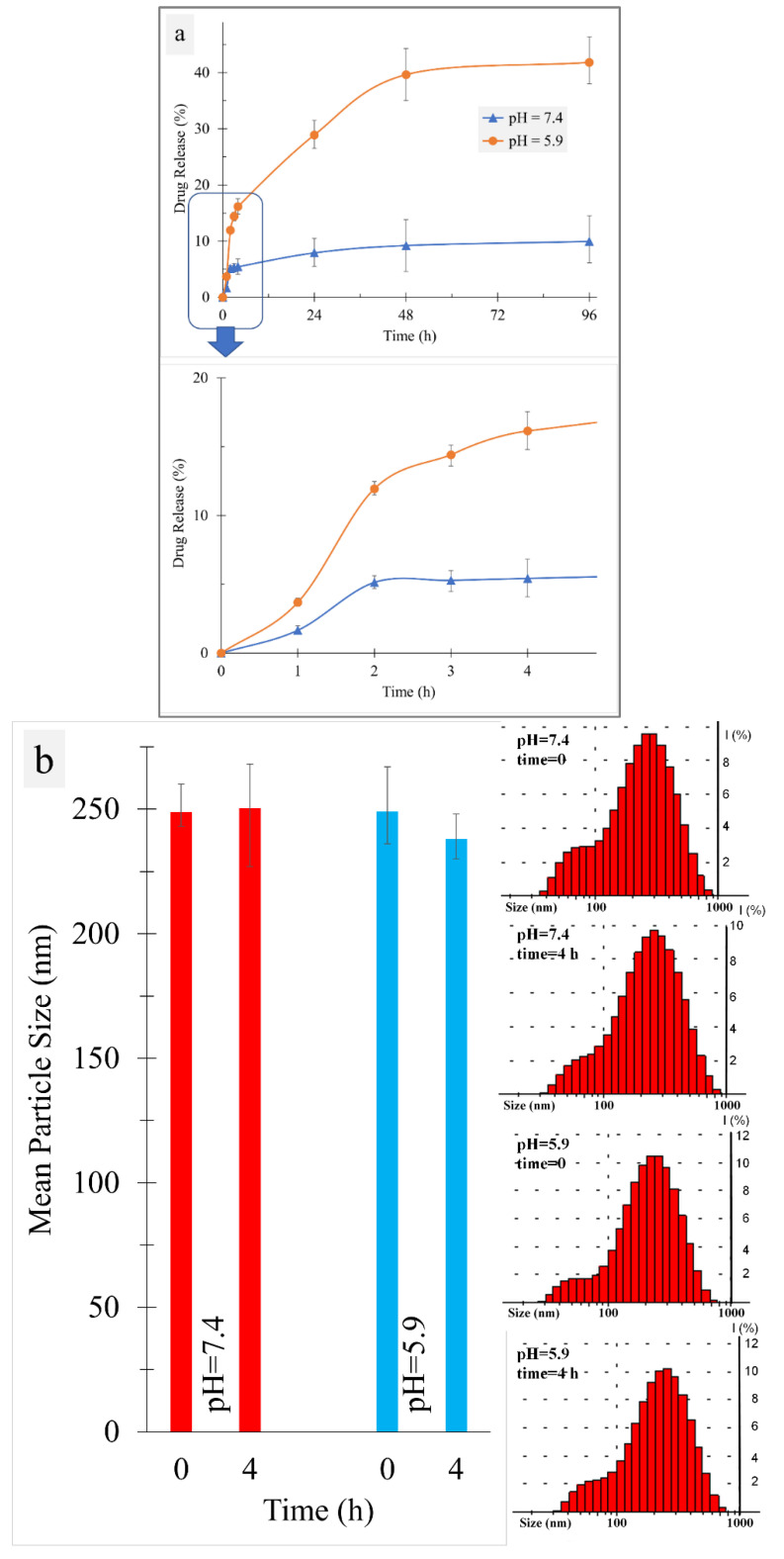
(**a**) Drug release kinetics at two different pH values of 5.9 (orange circle markers) and 7.4 (blue triangle markers) to mimic cancer cell and normal cell environments, respectively. Markers display the mean of readings for each time point of three independent specimens and error bars indicate the variation in the data. (**b**) Monitoring of the drug release process based on changes in particle size distribution and mean particle size.

The hydrazone bond, which was utilized for DOX loading, is known as a pH-sensitive agent. This characteristic is the key to designing smart materials, such as drug carriers [43]. In this work, two pH values (i.e., 7.4 and 5.9) were selected to mimic healthy cell and cancer cell environments, respectively [44]. Here, DOX should be released in cancer cells rather than normal cells. To assess this expectation, a drug release assay was accomplished and Figure 5a shows the obtained results conforming to the pH/time dependence of drug release. The kinetic release pattern in neutral acidity (pH = 7.4), which is a characteristic of healthy cells, was considerably slower and reached a plateau of about 10% after 96 h. Meanwhile, a higher rate of drug release in acidic pH, as a well-known trait of cancer cells, confirmed the smart behavior of the synthesized compound in the face of cancer cells. In this condition, a drug release of about 40% was observed after 24 h. In comparison with similar reported results, this achievement could be promising. This smart behavior mainly originates from the hydrazone bond [45]. Cleavage of this moiety in the synthesized compound is a pH control process and helped us in the design of this compound. The effect of pH on the hydrazone cleavage was evaluated by dynamics light scattering (DLS) to measure the mean particle diameter of the synthesized compound dissolved in 7.4 and 5.9 buffer solutions. Figure 5b shows the particle size distribution as well as the mean particle size of the specimens. Although particle size distributions in all of the samples were roughly similar, the mean particle size value was decreased after 4 h in the acidic pH, whereas no considerable changes were observed in the neutral acidity. This could support the previous discussion concerning the effects of hydrazone cleavage on drug release kinetics. Moreover, these results provided an estimate of the particle size of the synthesized compound. As shown in Figure 5b, the DLS results at t = 0 represent the TPE-PEGA-Hyd-DOX prodrug, which has an average particle size of 249 nm with the polydispersity index (pdi) of 0.29. In similar studies, this parameter is often lower. For instance, PEG-Pep-TPE/DOX nanoparticles were reported to have an average hydrodynamic diameter of 137 nm with the polydispersity index of 0.249 [29].

### 3.4. AIE Behavior

**Figure 6:**
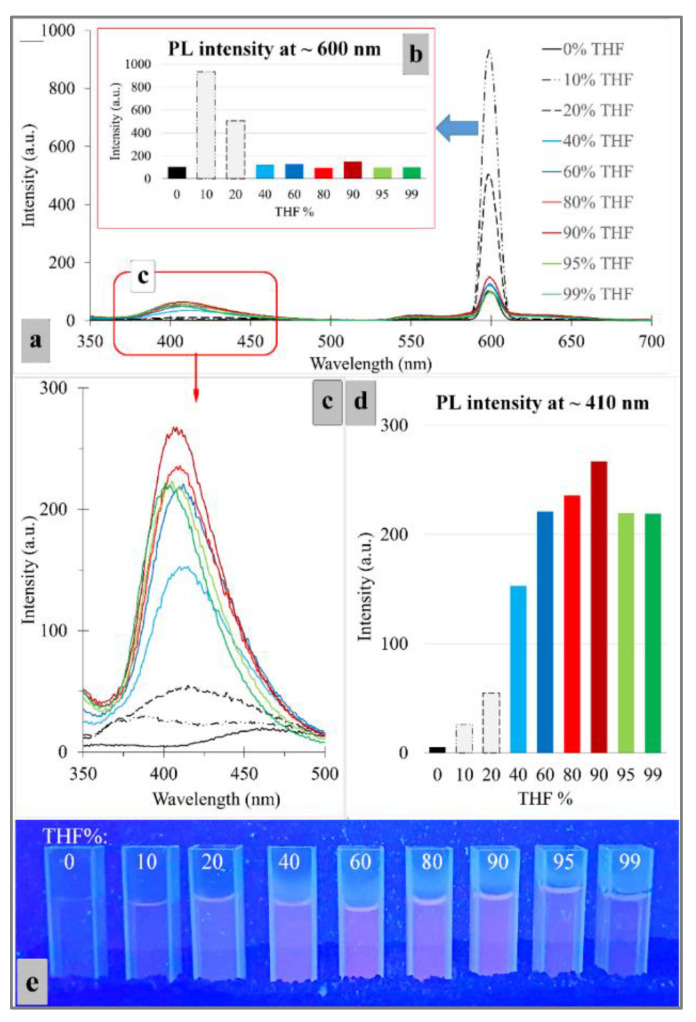
(**a**) The photoluminescence spectra of the prodrug with various H_2_O-THF ratios, excitation wavelength = 300 nm, PMT = 600 V. (**b**) The corresponding emission intensity values vs. THF percentages, in accordance with part (**a**,**c**) The photoluminescence spectra in the range of 350–500 nm focus on the AIE effect, excitation wavelength = 300 nm, PMT = 700 V. (**d**) The corresponding emission intensity values vs. THF percentages, in accordance with part (**c**,**e**) The relevant images of different THF percentages were captured using a UV lamp with a wavelength of 365 nm. The reason for using the excitation wavelength of 300 nm was to provide the necessary conditions for achieving simultaneous emission at ~400 and 600 nm from TPE and DOX moieties, respectively.

Due to the existence of TPE and DOX, the TPE-PEGA-Hyd-DOX compound was supposed to have AIE and fluorescence emission features. DOX is known as an intrinsic fluorescence chemical with an emission wavelength of ~600 nm [46] and TPEBIB has the AIE feature with an emission of ~400 nm [35]. The synthesized compound is soluble in water, whereas in organic solvents it is insoluble. Therefore, the H_2_O-THF mixture was used to evaluate its AIE trait. Figure 6a shows the photoluminescence spectra of the specimens with various H_2_O-THF ratios. These spectra were recorded under an excitation wavelength of 300 nm and photomultiplier tube (PMT) voltage of 600 V. Two major peaks were observed at ~400 and 600 nm, which are attributed to the emission of TPE and DOX, respectively. Figure 6b shows a bar chart to clarify the emission intensities in different THF percentages for DOX. The most intense fluorescence emission was observed in the mixture containing 10% THF, which shows the traditional ACQ effects. Figure 6c shows the photoluminescence spectra in the range of 350–500 nm, which were recorded under an excitation wavelength of 300 nm. Figure 6d illustrates that the maximum emission intensity was in the mixture containing 90% THF for TPEBIB, which shows a typical AIE feature. The ratiometric features for TPE-PEGA-Hyd-DOX can be observed on I_410 nm_/I_600 nm_. In the case of this compound, 90%-THF mixture reduced DOX emission, but showed a more intense effect of TPE emission. Therefore, the I_410 nm_/I_600 nm_ value increased. However, 10%-THF mixture led to a more intense effect of DOX emission, but reduced the TPE emission, thus the I_410 nm_/I_600 nm_ value decreased. This phenomenon might have originated from the fluorescence resonance energy transfer (FRET) between DOX and TPE [29]. Figure 6e shows the relevant images of different THF percentages captured using a UV lamp with a wavelength of 365 nm, showing the most emissive specimen in the 90%-THF mixture.

### 3.5. Cell Viability Assay and Drug Release Monitoring

**Figure 7:**
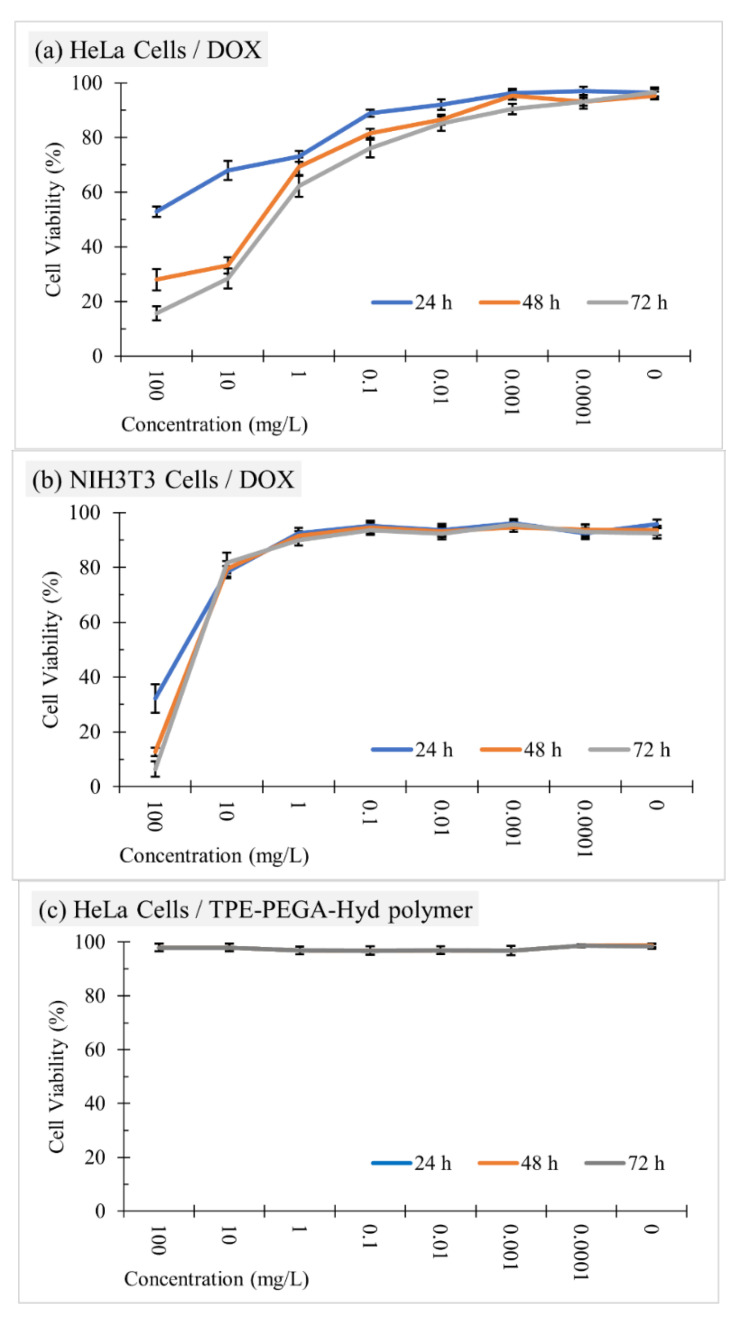

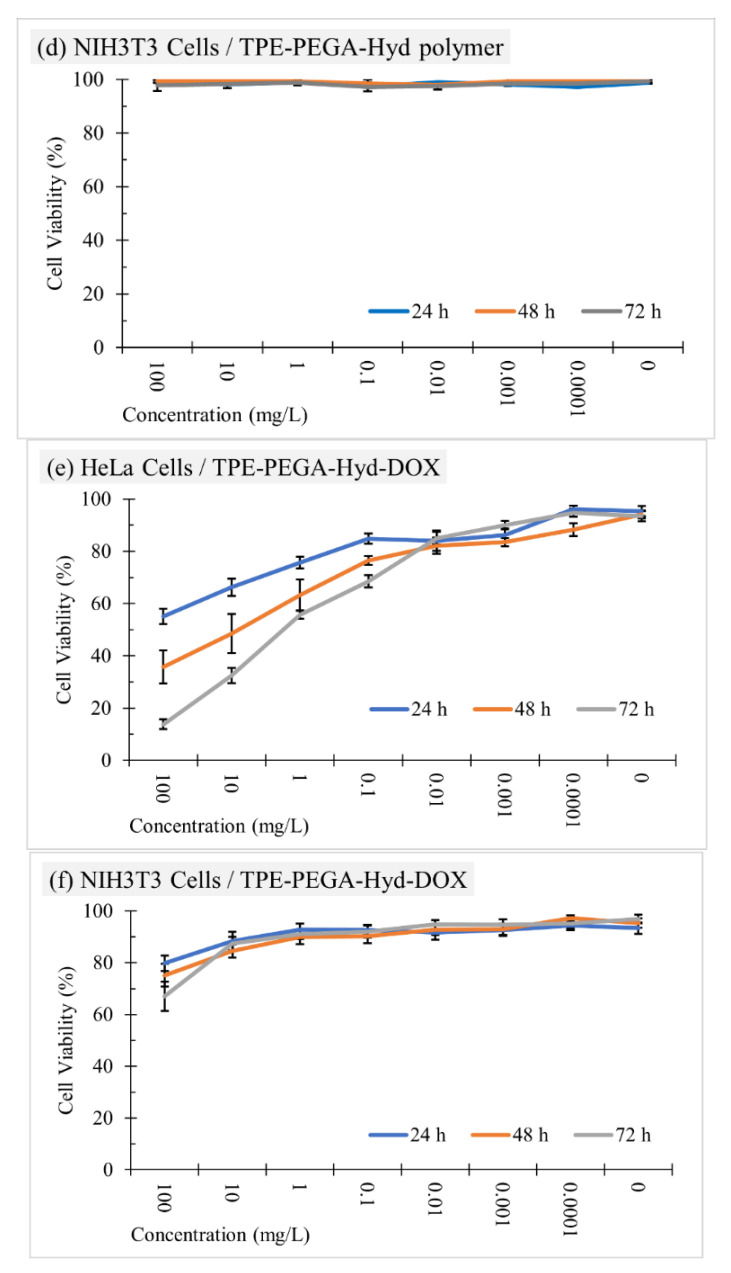
Cytotoxicity assay of the (**a**,**b**) pristine DOX, (**c**,**d**) TPE-PEGA-Hyd polymer, and (**e**,**f**) TPE-PEGA-Hyd-DOX prodrug in seven different concentrations against (**a**,**c**,**e**) HeLa and (**b**,**d**,**f**) NIH3T3 cells after 24, 48 and 72 h of incubation. Each data point was recorded from 12 repli-cates of a single microplate. The corresponding standard deviation values for different specimens based on 12 independent evaluations were presented in Table S1 and shown in the figure.

**Figure 8:**
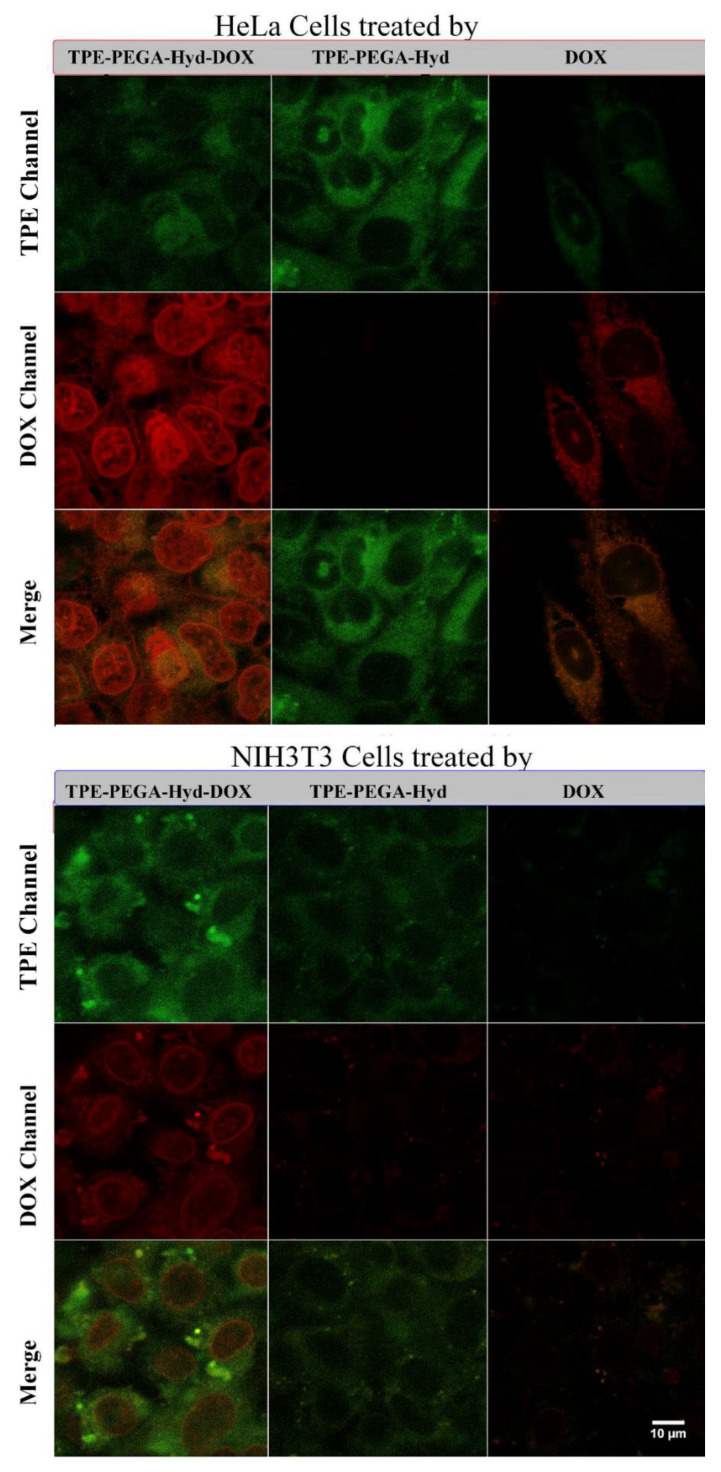
Confocal images of the HeLa and NIH3T3 cells treated by pristine DOX, TPE-PEGA-Hyd polymer, and TPE-PEGA-Hyd-DOX prodrug with a concentration of 10 mg/L after 6 and 24 h of incubation for HeLa and normal cells, respectively. The excitation wavelengths for DOX and AIE channels were 488 and 405 nm, whereas their emission wavelength ranges were 570–620 and 450–520 nm, respectively.

Figure 7 shows that the cell viability test results confirmed the high biocompatibility of the synthesized prodrug. This polymer exhibited no considerable effect against cancer or normal cells, where the corresponding death of normal or cancer cells was negligible even in the concentration of 100 mg/L. Moreover, the pristine DOX solution exhibited a concentration-dependent performance against the normal cells, whereas time also played a significant role in cancer cells. Normal cell viability was around 90% at the DOX concentration of up to 1 mg/L, then this parameter decreased to less than 30% at a concentration of 100 mg/L. The DOX solution showed a more intense effect against HeLa cells, where its influence was observed clearly even at a concentration of 0.01 mg/L. Moreover, the performance of DOX in cancer cell extermination was affected by the incubation time, where the longer the incubation time the higher the level of the cytotoxicity. However, this explanation aimed to clarify the effects of the polymer as well as an anticancer drug on the target cells. At present, the performance of the synthesized compound, TPE-PEGA-Hyd-DOX, against normal and cancer cells is supposed to be studied. The cytotoxicity of this compound against normal cells at a concentration of up to 1 mg/L was negligible, albeit reached cell viability of ~65% at a concentration of 100 mg/L after 72 h of incubation. A comparison between pristine DOX and TPE-PEGA-Hyd-DOX solutions in the same concentration confirmed that the conjugation of DOX to TPE-PEGA-Hyd polymer could control the drug release in normal cells and led to the protection of normal cells against DOX. This might originate from the nature of the hydrazone linker holding DOX, which could not be triggered in the neutral environment of normal cells. The main task of the synthesized compound is to prevent cancer cell survival and growth. Although the concentration of DOX in this compound is about 11% (in accordance with an experimental calculation), its performance in cancer cell killing is as strong as the pristine DOX solution at the same concentration. As an example, in a concentration of 100 mg/L, the amount of DOX in 100 mL of the DOX and TPE-PEGA-Hyd-DOX solutions are 10 and 1.1 mg, respectively. In this condition, the cytotoxicity levels of both compounds are similar. This might be related to the cellular uptake process. The anticancer drug needs to pass through cell walls to be effective in cell death. Loading DOX on the TPE-PEGA-Hyd polymer led to improving its uptake capability. Therefore, the cytotoxicity was improved albeit of the lower DOX concentration. To assess the cellular uptake, confocal microscopy provided us with some clues due to the specific feature of this compound. Figure 8 shows confocal images of healthy and cancer cells incubated with DOX, TPE-PEGA-Hyd polymer and TPE-PEGA-Hyd-DOX solutions, which would be utilized for intracellular drug tracking. Of note, the incubation time of healthy cells was 24 h, whereas 6 h of incubation was enough for the synthesized compound to reveal its effects on the cancer cells. However, Figure S9 shows confocal images of the cancer cells incubated for 24 h with DOX, TPE-PEGA-Hyd polymer and TPE-PEGA-Hyd-DOX solutions. In the case of healthy cells, the green emission in the TPE channel was observed using TPE-PEGA-Hyd polymer and TPE-PEGA-Hyd-DOX solutions, but not for DOX. The emission intensity in this channel illustrates the uptake process progression. Therefore, although the polymer uptake was not intensive, TPE-PEGA-Hyd-DOX solution uptake was accomplished at a detectable level. Moreover, the red emission in the DOX channel is an index showing drug release intensity. The moderate red emission, in this case, confirmed the low level of drug release in healthy cells. This observation could support the cytotoxicity discussions. In the case of cancer cells, the most important achievement of this work was observed. Pristine DOX solution uptake was not considerable, whereas the prodrug uptake was performed and a strong green emission could be observed. Moreover, a strong red emission in the DOX channel confirmed a promising level of drug release in the cytoplasm. The merged images of cancer cells clearly confirmed the high performance of the TPE-PEGA-Hyd-DOX compound in the viewpoints of cellular uptake and drug release. Cellular uptake is a surface-dependent phenomenon, whereas the high surface area is an indication of brush-like polymers. Therefore, a polymer brush compound could promote the cellular uptake procedure. This notable achievement using the AIE feature could open a new gate toward cancer therapy and drug delivery with high monitoring ability for in vivo intracellular drug tracking. One other considerable functional aspect of this research relates to the releasing location of DOX. In Figure 8, it has been shown that pristine DOX caused the cytoplasm to be emissive, whereas the prodrug made the cell nucleus emissive. This indicated that drug-releasing occurred in the cell nuclei, where DOX exhibits the highest performance in killing cancer cells.

## 4. Conclusions

In this work, a brush-like polymer with AIE features for drug delivery has been designed and synthesized based on the Cu^0^-mediated reversible-deactivation radical polymerization (RDRP) method. TPEBIB with AIE features was synthesized and used as an initiator in the RDRP. Hydrazone bond as a tool for controlling drug release was added to the system using tert-butyl-2-acryloylhydrazine-1-carboxylate as a monomer in the RDRP. The brush-like structure of the targeted compound provides a means for loading different types of drugs and targeting agents. This feature was provided utilizing PEGA as another monomer in the RDRP. DOX, as an anticancer drug with florescence behavior, was used to complete the targeted compound. The smart prodrug, named TPE-PEGA-Hyd-DOX, has been successfully synthesized, which contains ~11 wt% of DOX. This is partially stable in normal cell biosystems, but releases DOX in cancer cell environments due to the hydrazone cleavage in acidic pH. This prodrug in H_2_O-THF mixture exhibits emission at two different wavelength ranges, 380–440 nm (resulting from TPE) and 580–620 nm (resulting from DOX) with a ratiometric feature on I_410 nm_/I_600 nm_. The synthesized polymer (TPE-PEGA-Hyd) is completely biocompatible against both normal and cancer cells, whereas cytotoxicity of the polymer is a pH-dependent parameter. This compound is partially passive in healthy cell environments and strongly active in typical cancer cells. The use of pristine DOX leads to the accumulation of drugs in the cytoplasm of cancer cells, whereas taking advantage of the prodrug leads to the release of DOX moieties in the nuclei of cancer cells. The emission of the prodrug in the TPE and DOX channels provides the capability of accurate monitoring of cellular uptake and drug release in vitro or in vivo.

## Data Availability

Exclude this statement.

## Supplementary Materials

The following supporting information can be downloaded at: https://www.mdpi.com/article/10.3390/bios12060373/s1, Figure S1: ^1^H-NMR spectrum of the synthesized TPEOH; Figure S2: ^1^H-NMR spectrum of the synthesized TPEBIB; Figure S3: Synthesis pathway of TPEBIB. Firstly, TPEOH was synthesized using benzophenone and 4-hydroxybenzophenone, then TPEBIB was prepared by adding BIBB to the TPEOH compound; Figure S4: ^1^H-NMR spectrum of the synthesized hydrazine monomer; Figure S5: ^13^C-NMR spectrum of the synthesized hydrazine monomer; Figure S6: Synthesis pathway of the hydrazine monomer using acrylic acid and tert-butyl carbazate; Figure S7: The calibration curve of DOX in PBS. As the R2 value is very close to 1, the relation between parameters x and y is a positive linear relationship, so the line equation could be used for measuring DOX concentration according to the corresponding absorbance value; Figure S8: Schematic illustration of the synthesized brush-like polymer. According to the 1H-NMR result in Figure 2a, the polymerization degree or n is 21, in which 19 units of PEGA side chain end and 2 units of hydrazine end exist in each polymer chain; Figure S9: Confocal images of the HeLa cells treated by pristine DOX, TPE-PEGA-Hyd polymer, and TPE-PEGA-Hyd-DOX pro-drug with a concentration of 10 mg/L after 24 h of incubation. The excitation wavelengths for DOX and AIE channels were 488 and 405 nm, whereas their emission wavelength ranges were 570–620 and 450–520 nm, respectively. In comparison with Figure 8, the effects of the synthesized prodrug on cancer cells are more severe and significant; Figure S10: The Nuclei of the cells have been labelled using an image analyser. This is a simple way illustrating the exact loca-tion of drug release, which has been targeted by the carrier; Table S1: Numerical data of the Cytotoxicity assay.
