# Supplementary material for: Brush-like Polymer Prodrug with Aggregation-Induced Emission Features for Precise Intracellular Drug Tracking
Source: Biosensors (Basel). 2022 May 29;12(6):373. doi: 10.3390/bios12060373 (PMC9221197; doi:10.3390/bios12060373)
Supplement: Supplementary file 1 [file biosensors-12-00373-s001.zip › biosensors-1637306-supplementary.pdf]

Electronic Supplementary Information (ESI)

Brush-Like Polymer Prodrug with Aggregation-Induced Emission Features for Precise Intracellular Drug Tracking

Sanaz Naghibi [1], Soheila Sabouri [2], Yuning Hong [2, 3], Zhongfan Jia [1, *], Youhong Tang [1, 3,*]

1. Institute for NanoScale Science and Technology, College of Science and Engineering, Flinders University, Tonsley, SA 5042, Australia; sanaz.naghibi@flinders.edu.au (S.N.)

2. Department of Biochemistry and Chemistry, La Trobe Institute for Molecular Science, La Trobe Univer-sity, Bundoora, VIC 3086, Australia; s.sabouri@latrobe.edu.au (S.S.); y.hong@latrobe.edu.au (Y.H.)

3. Australia-China Joint Research Centre on Personal Health Technologies, Tonsley, SA 5042, Australia

* Correspondence: zhongfan.jia@flinders.edu.au (Z.J.); youhong.tang@flinders.edu.au (Y.T.);

Tel.: +61-8-8201-2804 (Z.J.); +61-8-8201-2138 (Y.T.)

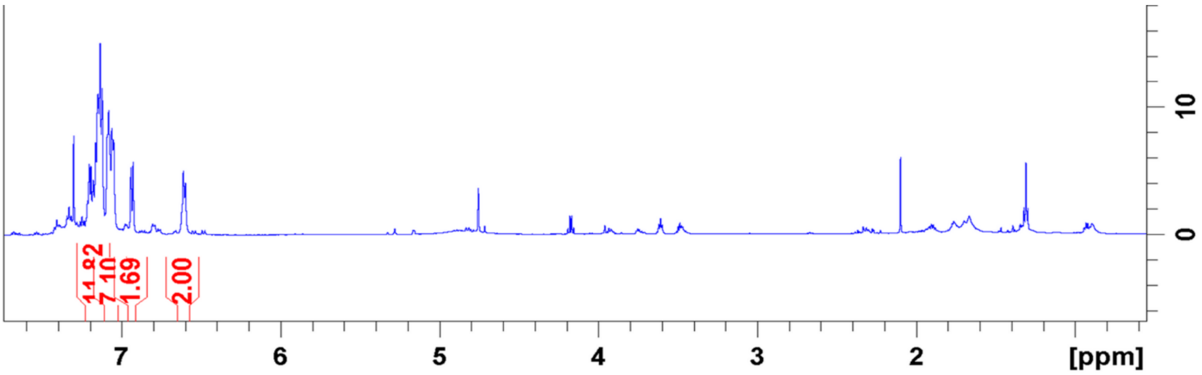

Figure S1. ^{1}H-NMR spectrum of the synthesized TPEOH.

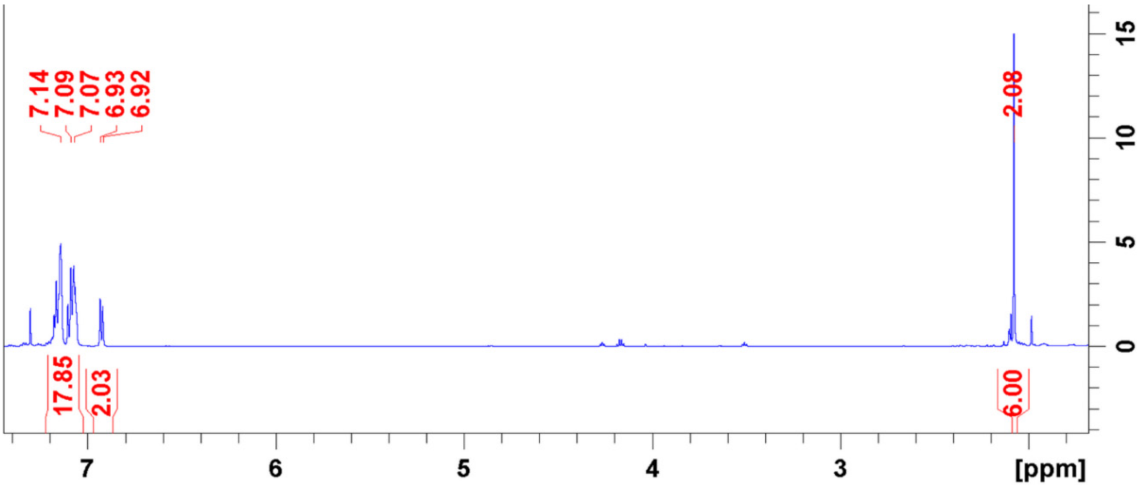

Figure S2. ^{1}H-NMR spectrum of the synthesized TPEBIB.

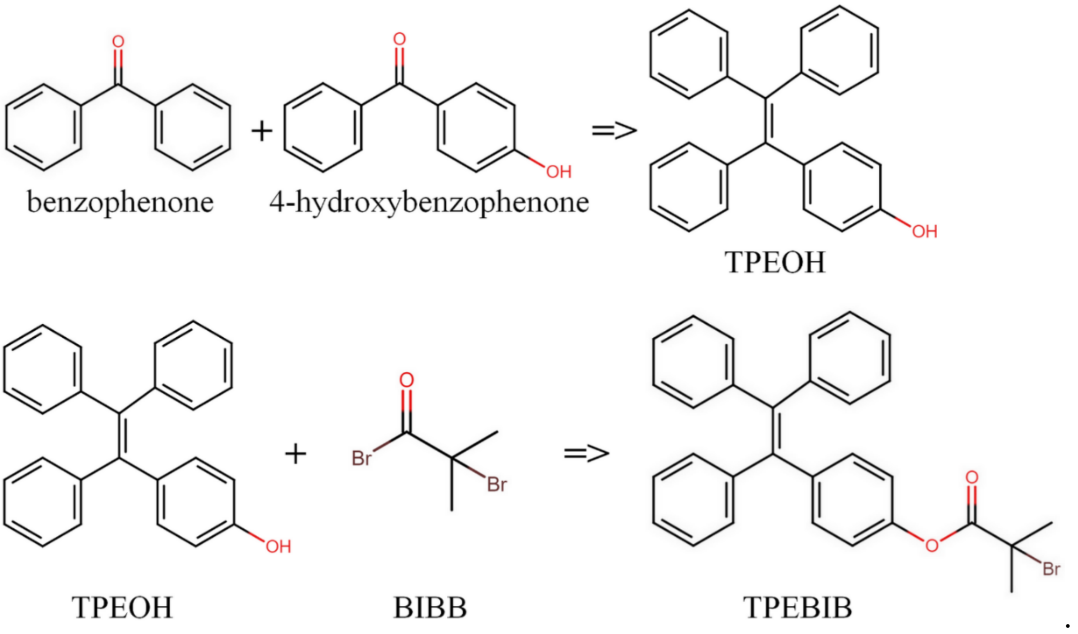

Figure S3. Synthesis pathway of TPEBIB. Firstly, TPEOH was synthesized using benzophenone and 4-hydroxybenzophenone, then TPEBIB was prepared by adding BIBB to the TPEOH compound.

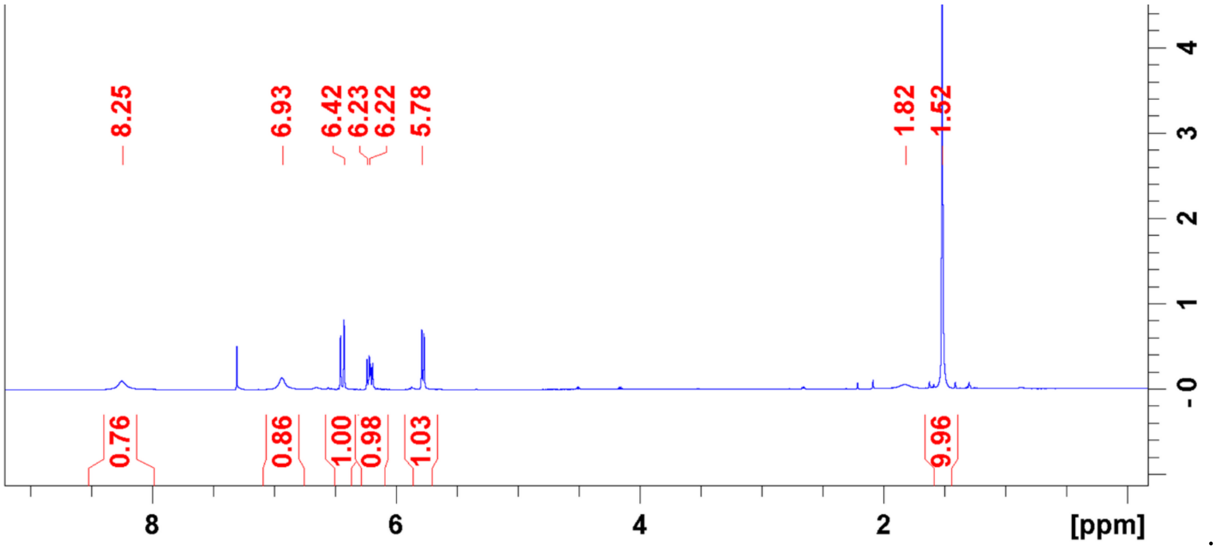

Figure S4. ^{1}H-NMR spectrum of the synthesized hydrazine monomer.

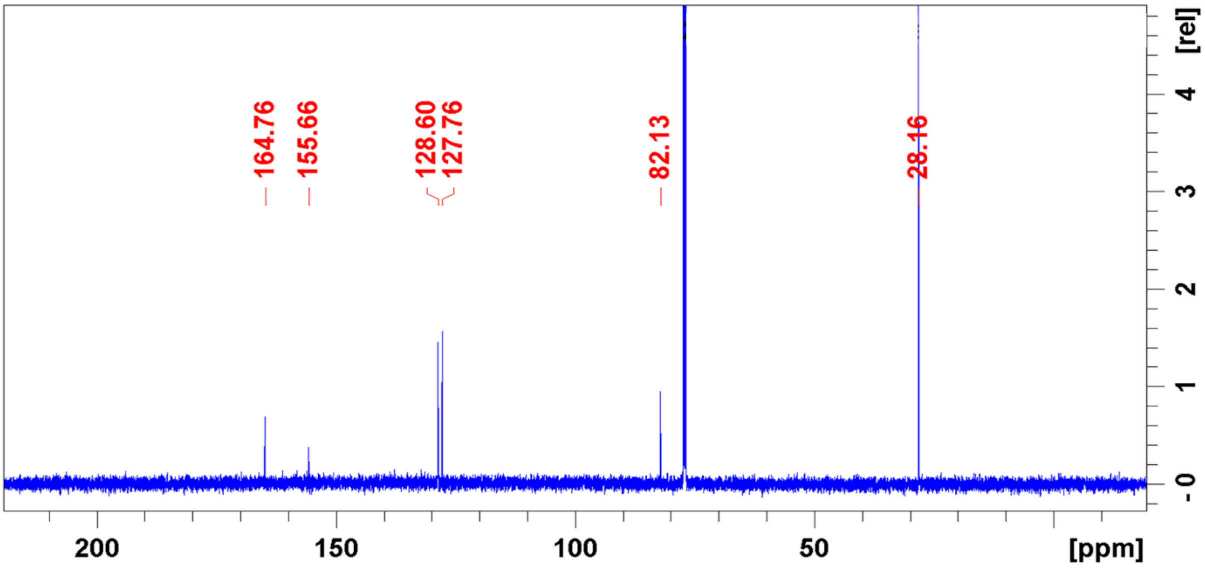

Figure S5. ^{13}C-NMR spectrum of the synthesized hydrazine monomer.

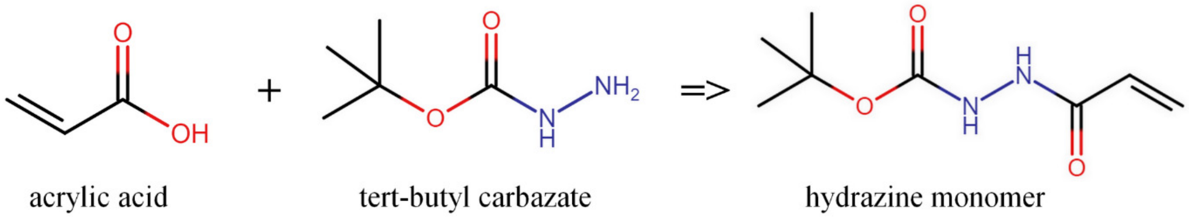

Figure S6. Synthesis pathway of the hydrazine monomer using acrylic acid and tert-butyl carbazate.

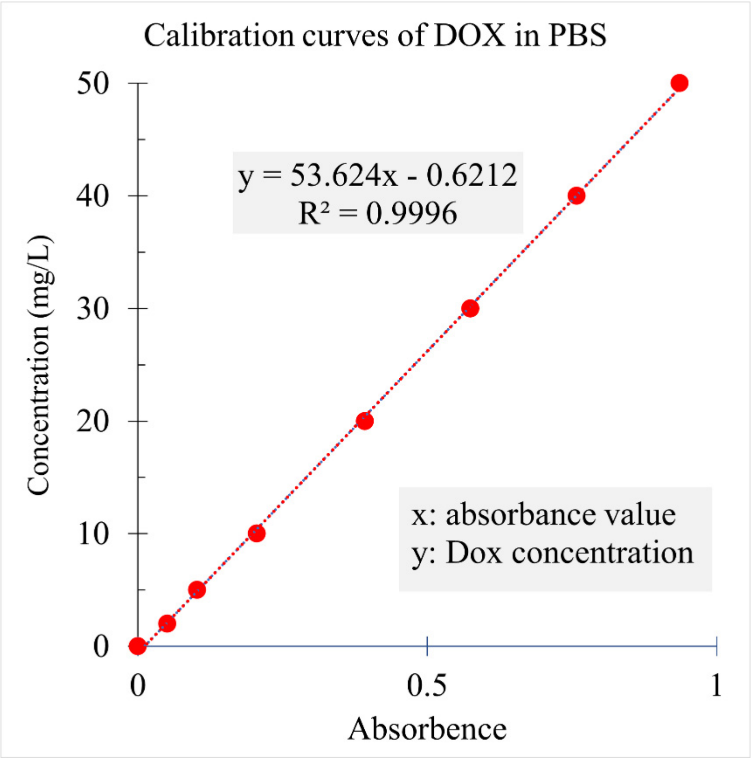

Figure S7. The calibration curve of DOX in PBS. As the R2 value is very close to 1, the relation between parameters x and y is a positive linear relationship, so the line equation could be used for measuring DOX concentration according to the corresponding absorbance value.

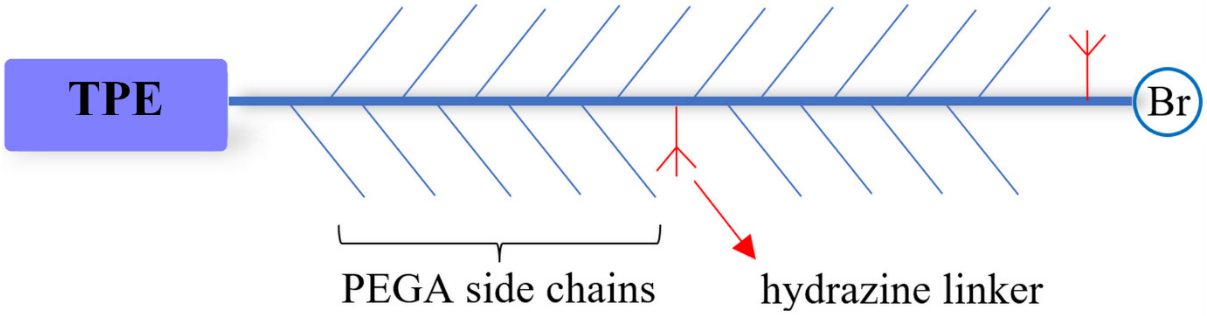

Figure S8. Schematic illustration of the synthesized brush-like polymer. According to the 1H-NMR result in Figure 2(a), the polymerization degree or n is 21, in which 19 units of PEGA side chain end and 2 units of hydrazine end exist in each polymer chain.

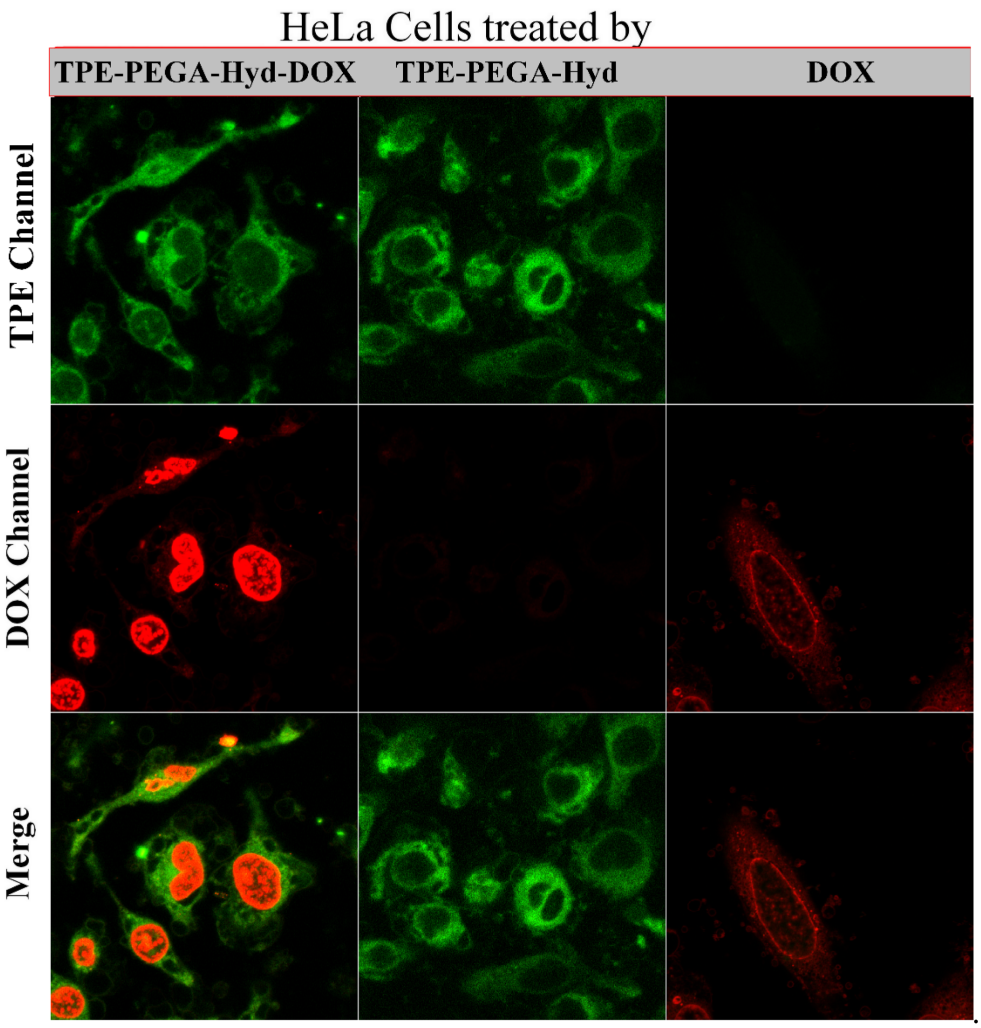

Figure S9. Confocal images of the HeLa cells treated by pristine DOX, TPE-PEGA-Hyd polymer, and TPE-PEGA-Hyd-DOX prodrug with a concentration of 10 mg/L after 24 h of incubation. The excitation wavelengths for DOX and AIE channels were 488 and 405 nm, whereas their emission wavelength ranges were 570-620 and 450-520 nm, respectively. In comparison with Figure 8, the effects of the synthesized prodrug on cancer cells are more severe and significant.

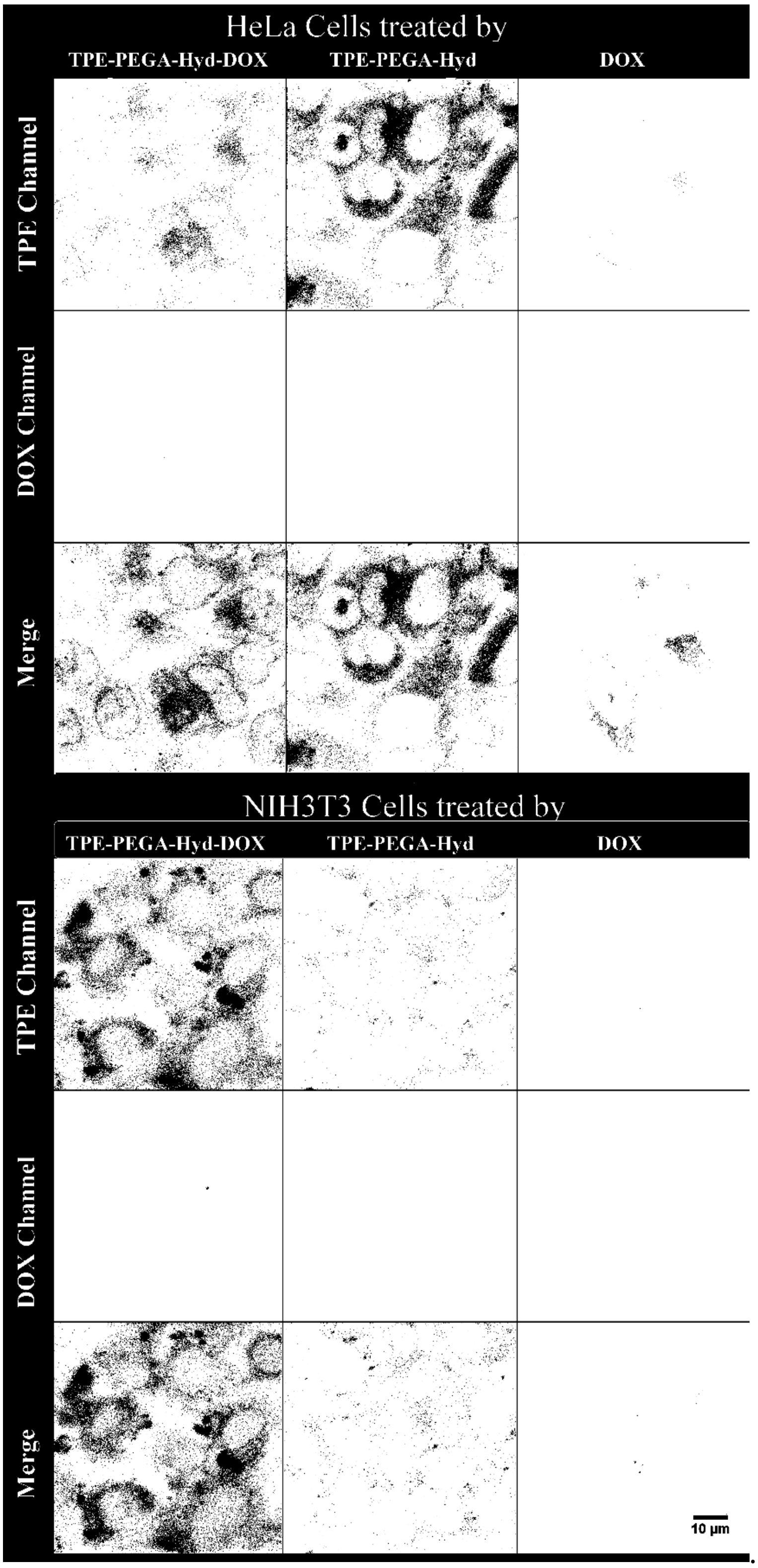

Figure S10. The Nuclei of the cells have been labelled using an image analyser. This is a simple way illustrating the exact location of drug release, which has been targeted by the carrier.

Table S1. Numerical data of the Cytotoxicity assay. Figure 7 has been prepared according to these data. Each Table represents readings for each data point of 12 independent samples, average, and standard deviation value, which is calculated using $\sigma = \sqrt{\frac{\Sigma(Xi-\mu)^2}{N}}$, where σ, Xi, μ, and N are standard deviation, each value from the population, the population mean and the size of the population, respectively. Average values have been utilized for drawing graphs of Figure 7.

Sample	HeLa cell / DOX, Figure 7a							
Time	24 h							
C (mg/L)	100	10	1	0.1	0.01	0.001	0.0001	0
1	50	80	75.75758	87.5	93.54839	100	96.2963	92.59259259
2	55.55556	80.64516	75	89.65517	90	96.15385	93.54839	96.875
3	52.17391	72.22222	76.19048	91.66667	89.28571	94.59459	100	97.22222222
4	58.62069	71.42857	75	88.88889	85.18519	93.10345	100	97.05882353
5	57.57576	66.66667	68.42105	84.375	89.28571	95.12195	96.9697	96.66666667
6	50	65	78.26087	85.29412	93.33333	97.36842	100	96.96969697
7	56	61.53846	76.92308	93.33333	100	90.90909	100	97.2972973
8	48.3871	60.86957	72.97297	90.32258	94.44444	93.02326	91.17647	100
9	51.42857	64.51613	74.19355	87.09677	93.54839	100	96.66667	92.10526316
10	51.51515	60	66.66667	91.17647	96.2963	100	93.93939	100
11	55.88235	69.56522	70.83333	87.87879	89.47368	97.22222	100	92.85714286
12	47.05882	63.15789	66.66667	90	89.65517	97.4359	95	96.875
μ	52.84983	67.96749	73.07385	88.93232	92.00469	96.24439	96.96641	96.37664211
σ	3.766	6.999	4.001	2.629	3.955	2.995	3.086	2.597

Sample	HeLa cell / DOX, Figure 7a							
Time	48 h							
C (mg/L)	100	10	1	0.1	0.01	0.001	0.0001	0
1	20	33.33333	62.5	78.125	88.23529	91.42857	91.30435	100
2	33.33333	19.35484	62.96296	84	80.64516	97.14286	94.73684	93.54839
3	28.57143	27.77778	69.56522	84.375	88.23529	89.74359	96.2963	96.875
4	33.33333	28.57143	66.66667	82.14286	84.375	93.54839	92.59259	91.42857
5	36.84211	33.33333	64.70588	80.95238	86.04651	95.12195	95.45455	93.93939
6	18.18182	35	68	85.18519	92.68293	97.36842	92.59259	97.14286
7	25	38.46154	66.66667	78.26087	90.90909	96.66667	96.42857	96.66667
8	28.57143	39.13043	73.91304	82.75862	84.61538	94.59459	89.65517	93.10345
9	28	35.48387	60.86957	82.75862	88.23529	92.30769	96	97.22222
10	42.10526	40	79.41176	74.28571	87.87879	100	92.59259	90.625
11	39.13043	30.43478	75	84.375	85.71429	96.66667	88.46154	96.875
12	40.90909	36.84211	81.81818	81.25	80	100	90.47619	96.55172
μ	31.16485	33.14362	69.34	81.5391	86.46442	95.38245	93.04927	95.33152
σ	7.830103	5.885045	6.78842	3.222307	3.741752	3.218151	2.732985	2.765909

Sample	HeLa cell / DOX, Figure 7a							
Time	72 h							
C (mg/L)	100	10	1	0.1	0.01	0.001	0.0001	0
1	11.76471	24.32432	59.25926	72	80.64516	89.28571	86.36364	95.83333
2	5.555556	26.47059	48.27586	76	77.41935	88.57143	92.85714	100
3	20	27.5	64.28571	85	88.46154	97.2973	92.85714	95.2381
4	21.42857	30.76923	67.85714	84	93.54839	85.29412	92	96.42857
5	16.66667	27.58621	62.5	75	89.74359	90	86.36364	100
6	17.3913	21.62162	77.77778	80.76923	89.74359	88.88889	95.65217	100
7	11.11111	36	72	65.21739	85.29412	88.46154	94.73684	90
8	18.18182	33.33333	58.33333	70	82.05128	90.32258	85.18519	100
9	10	45.45455	64	84.21053	86.11111	93.54839	100	91.30435
10	23.80952	22.22222	62.96296	68	89.74359	90	95.45455	100
11	17.3913	19.35484	52.17391	76	81.81818	86.48649	100	95.65217
12	14.28571	25	58.62069	76.19048	76.47059	96.875	95	93.54839
μ	15.63219	28.30308	62.33722	76.0323	85.08754	90.41929	93.03919	96.50041
σ	5.255084	7.247245	8.031536	6.5258	5.408678	3.714153	4.938272	3.592512

Sample	NIH3T3 cell / DOX, Figure 7b							
Time	24 h							
C (mg/L)	100	10	1	0.1	0.01	0.001	0.0001	0

1	36.84211	80.55556	89.28571	96.9697	82.05128	93.75	94.59459	100
2	56.25	79.54545	96.2963	94.73684	88.37209	95.34884	91.17647	95.74468
3	29.62963	70.96774	94.11765	94.87179	93.33333	94.59459	84.84848	97.4359
4	23.07692	79.54545	87.5	92.85714	97.4359	97.67442	96.66667	89.74359
5	33.33333	81.57895	87.80488	97.22222	93.93939	100	94.59459	94.87179
6	25	86.66667	92.85714	86.48649	93.75	92.68293	95	92.85714
7	37.5	78.26087	93.93939	100	95.34884	95.12195	92.5	91.42857
8	26.08696	77.5	96.15385	94.59459	97.22222	97.67442	93.18182	100
9	30.76923	71.15385	96.9697	97.36842	96.9697	97.61905	87.80488	97.36842
10	42.85714	77.77778	96.55172	92.68293	100	97.82609	92.10526	97.4359
11	16.66667	75	87.17949	97.22222	89.3617	90.625	96.875	95.34884
12	27.77778	81.39535	91.17647	96.9697	93.93939	100	90.47619	96.9697
μ	32.14915	78.32897	92.48602	95.16517	93.47699	96.07644	92.48533	95.76704
σ	10.37832	4.422539	3.770777	3.445796	4.880549	2.886007	3.549416	3.161782

Sample	NIH3T3 cell / DOX, Figure 7b							
Time	48 h							
C (mg/L)	100	10	1	0.1	0.01	0.001	0.0001	0
1	9.52381	84.44444	88.37209	92.30769	85.29412	100	91.42857	91.11111
2	12.5	71.73913	95.12195	90.2439	96.42857	97.87234	97.22222	92.10526
3	13.33333	85.36585	93.61702	94.59459	88.57143	95.55556	97.36842	88.88889
4	14.70588	83.09859	94.59459	97.2973	94.59459	97.5	95	90.47619
5	10.71429	79.66102	96.9697	100	92.10526	92.5	87.5	88.63636
6	11.76471	70.90909	91.11111	91.17647	92.85714	92.10526	92.30769	92.30769
7	11.11111	83.78378	92.30769	97.22222	97.14286	93.18182	84.78261	94.11765
8	21.05263	86.36364	88.23529	95.45455	96.9697	88.63636	91.42857	94.59459
9	13.63636	79.06977	84.09091	97.4359	91.17647	95.74468	94.44444	95.74468
10	13.33333	79.24528	92.68293	97.2973	90.625	95.65217	97.22222	100
11	12.5	79.48718	89.74359	92.10526	94.59459	97.61905	97.4359	96.875
12	8.333333	70	90.625	88.09524	97.36842	89.74359	97.36842	96.9697
μ	12.70907	79.43065	91.45599	94.43587	93.14401	94.6759	93.62576	93.48559
σ	3.191557	5.734815	3.547873	3.612134	3.782451	3.469986	4.243486	3.508814

Sample	NIH3T3 cell / DOX, Figure 7b							
Time	72 h							
C (mg/L)	100	10	1	0.1	0.01	0.001	0.0001	0
1	6.25	90.90909	92.5	91.42857	89.47368	92.68293	87.5	88
2	0	80	90.90909	97.36842	93.54839	92.10526	100	97.22222
3	0	88.23529	87.09677	95.12195	92.59259	94.87179	96.875	93.87755
4	10.52632	85.71429	91.42857	88.37209	86.11111	96.9697	97.4359	88.09524
5	6.451613	78.26087	87.5	92.10526	92.30769	97.22222	92.5	84.09091
6	0	78.94737	92.5	93.93939	86.48649	96.875	87.5	95
7	10.34483	77.77778	94.73684	97.2973	94.59459	94.73684	91.42857	93.18182
8	15.625	84.21053	85.36585	89.74359	95.12195	89.74359	81.57895	97.67442
9	13.7931	61.11111	82.97872	90.625	97.4359	97.14286	92.59259	95
10	8.695652	85.71429	94.44444	93.93939	100	97.4359	93.54839	92.85714
11	0	82.35294	91.17647	94.59459	88.37209	97.5	96.875	93.61702
12	5.263158	86.36364	87.5	97.2973	91.17647	100	97.14286	90.69767
μ	6.412472	81.6331	89.84473	93.48607	92.26841	95.60717	92.91477	92.44283
σ	5.582216	7.685595	3.697114	3.058878	4.235202	2.878588	5.307894	4.032659

Sample	HeLa cell / TPE-PEGA-Hyd polymer, Figure 7c							
Time	24 h							
C (mg/L)	100	10	1	0.1	0.01	0.001	0.0001	0
1	93.75	96.15385	93.75	93.75	96.77419	91.48936	97.14286	100
2	96.66667	93.33333	93.10345	92.59259	97.5	97.82609	97.22222	96.77419
3	100	100	100	100	95.45455	90.625	98.50746	100
4	100	100	96.9697	97.4359	100	92.64706	100	98.46154
5	100	96.9697	91.89189	90.32258	93.18182	100	98.7013	97.01493
6	100	93.18182	97.5	96.9697	92.68293	100	97.05882	98.52941
7	100	100	96.2963	97.36842	96.66667	96.49123	100	97.22222
8	96.875	100	100	100	100	97.72727	100	98.4127
9	100	94.59459	100	100	93.10345	100	98.64865	98.30508

10	94.11765	100	100	100	97.61905	100	97.10145	100
11	93.18182	100	97.77778	97.91667	100	98	100	100
12	100	100	94.87179	95.12195	100	96.61017	98.61111	100
μ	97.88259	97.85277	96.84674	96.78982	96.91522	96.78468	98.58282	98.72667
σ	2.815994	2.841241	2.914491	3.229685	2.82032	3.4115	1.224752	1.261239

Sample	HeLa cell / TPE-PEGA-Hyd polymer, Figure 7c							
Time	48 h							
C (mg/L)	100	10	1	0.1	0.01	0.001	0.0001	0
1	93.75	96.15385	93.75	93.75	96.77419	91.48936	97.22222	100
2	96.66667	93.33333	93.10345	92.59259	97.5	97.82609	97.2973	96.875
3	100	100	100	100	95.45455	90.625	98.52941	100
4	100	100	96.9697	97.4359	100	92.64706	100	98.48485
5	100	96.9697	91.89189	90.32258	93.18182	100	98.71795	97.10145
6	100	93.18182	97.5	96.9697	92.68293	100	97.14286	98.55072
7	100	100	96.2963	97.36842	96.66667	96.49123	100	97.2973
8	96.875	100	100	100	100	97.72727	100	98.4375
9	100	94.59459	100	100	93.10345	100	98.66667	98.33333
10	94.11765	100	100	100	97.61905	100	97.1831	100
11	93.18182	100	97.77778	97.91667	100	98	100	100
12	100	100	94.87179	95.12195	100	96.61017	98.63014	100
μ	97.88259	97.85277	96.84674	96.78982	96.91522	96.78468	98.6158	98.75668
σ	2.815994	2.841241	2.914491	3.229685	2.82032	3.4115	1.190395	1.226115

Sample	HeLa cell / TPE-PEGA-Hyd polymer, Figure 7c							
Time	72 h							
C (mg/L)	100	10	1	0.1	0.01	0.001	0.0001	0
1	93.75	96.15385	93.75	93.75	96.77419	91.48936	97.22222	100
2	96.66667	93.33333	93.10345	92.59259	97.5	97.82609	97.2973	96.875
3	100	100	100	100	95.45455	90.625	98.52941	100
4	100	100	96.9697	97.4359	100	92.64706	100	98.48485
5	100	96.9697	91.89189	90.32258	93.18182	100	98.71795	97.10145
6	100	93.18182	97.5	96.9697	92.68293	100	97.14286	98.55072
7	100	100	96.2963	97.36842	96.66667	96.49123	100	97.2973
8	96.875	100	100	100	100	97.72727	100	98.4375
9	100	94.59459	100	100	93.10345	100	98.66667	98.33333
10	94.11765	100	100	100	97.61905	100	97.1831	100
11	93.18182	100	97.77778	97.91667	100	98	100	100
12	100	100	94.87179	95.12195	100	96.61017	98.63014	94.54545
μ	97.88259	97.85277	96.84674	96.78982	96.91522	96.78468	98.6158	98.30213
σ	2.815994	2.841241	2.914491	3.229685	2.82032	3.4115	1.190395	1.658207

Sample	NIH3T3 cell / TPE-PEGA-Hyd polymer, Figure 7d							
Time	24 h							
C (mg/L)	100	10	1	0.1	0.01	0.001	0.0001	0
1	100	100	100	91.30435	97.82609	100	98.48485	100
2	96.77419	97.22222	100	100	100	100	100	100
3	100	100	100	100	100	97.91667	100	100
4	100	97.4359	97.5	100	100	100	100	98.21429
5	100	97.5	100	100	89.74359	100	100	100
6	100	100	100	100	100	100	100	100
7	100	100	100	94.87179	100	100	98.4127	100
8	100	100	95.34884	100	95.12195	95	96.8254	100
9	100	100	100	97.36842	100	98.07692	100	95.91837
10	96	100	100	97.5	100	100	100	100
11	100	100	100	100	98.07692	100	100	98.48485
12	100	100	97.4359	100	95.65217	100	100	100
μ	99.39785	99.34651	99.19039	98.42038	98.03506	99.24947	99.47691	99.38479
σ	1.415974	1.183834	1.554828	2.782178	3.162065	1.545636	1.027347	1.264998

Sample	NIH3T3 cell / TPE-PEGA-Hyd polymer, Figure 7d						
Time	48 h						
C (mg/L)	100	10	1	0.1	0.01	0.001	0.0001	0
1	100	97.36842	97.56098	100	100	100	95	100
2	100	100	97.5	100	100	97.14286	92.64706	98.11321
3	97.67442	97.2973	100	97.4359	100	94.87179	100	100
4	100	95	94.11765	100	100	100	94.44444	98.4127
5	95.45455	95.45455	100	94.59459	100	92.59259	98.11321	100
6	100	100	100	97.5	100	100	96.66667	100
7	100	100	100	90	100	100	100	97.01493
8	100	100	97.2973	97.5	97.22222	100	91.07143	100
9	97.22222	100	100	97.2973	95.45455	100	100	93.22034
10	100	100	100	97.14286	97.77778	100	100	98
11	100	95.12195	100	95.65217	95.83333	94.64286	100	100
12	100	97.61905	100	100	100	97.67442	98.18182	100
μ	99.19593	98.15511	98.87299	97.26024	98.85732	98.07704	97.17705	98.7301
σ	1.538217	2.100035	1.879647	2.901234	1.783971	2.681004	3.190865	2.036087

Sample	NIH3T3 cell / TPE-PEGA-Hyd polymer, Figure 7d							
Time	72 h							
C (mg/L)	100	10	1	0.1	0.01	0.001	0.0001	0
1	100	100	100	100	100	100	100	100
2	100	100	96.2963	100	97.4359	100	100	98.27586
3	100	100	100	100	92.5	100	100	100
4	95	94.59459	100	92.68293	100	97.22222	98.14815	100
5	100	97.4359	100	94.87179	97.61905	97.4359	100	96.49123
6	100	100	94.59459	97.36842	100	97.36842	100	98.14815
7	85.29412	100	96.66667	100	94.87179	94.87179	95.91837	100
8	100	93.33333	100	92.10526	97.4359	100	90.56604	100
9	96.42857	100	100	100	100	100	100	100
10	100	100	97.2973	100	96.55172	100	100	100
11	100	93.10345	100	96.66667	94	97.91667	100	98.24561
12	96.66667	100	100	92.10526	100	96.22642	96.22642	100
μ	97.78245	98.20561	98.7379	97.15003	97.53453	98.42012	98.40491	99.2634
σ	4.328748	2.847035	1.95952	3.371809	2.633953	1.812639	2.904496	1.178616

Sample	HeLa cell / TPE-PEGA-Hyd-DOX prodrug, Figure 7e							
Time	24 h							
C (mg/L)	100	10	1	0.1	0.01	0.001	0.0001	0
1	63.7325	75	71.875	85.71429	80	88.88889	92.85714	100
2	55.57692	65.38462	74.35897	82.35294	75	80.95238	100	87.09677
3	59.13043	69.56522	73.80952	78.78788	84.61538	83.72093	100	92.30769
4	53.83333	63.33333	68.42105	88.88889	82.85714	88.88889	96.2963	100
5	57.7654	68	77.5	88.88889	75.55647	91.30435	96.15385	96.2963
6	51.93333	63.33333	76.47059	85.71429	84.61538	91.30435	96.42857	94.11765
7	50.64706	61.76471	80.55556	82.35294	80	88.88889	93.10345	96.42857
8	48.6875	59.375	70.27027	82.35294	63.63636	88.88889	93.10345	96.15385
9	64.17391	78.26087	75.67568	78.78788	67.14286	78.04878	96.2963	100
10	58.57143	71.42857	76.31579	85.71429	63.63636	88.88889	100	93.93939
11	50.78947	63.15789	77.5	88.88889	80	86.36364	96.42857	90.625
12	45.55556	55.55556	85.29412	88.88889	69.56522	78.04878	92.59259	96.875
μ	55.03307	66.17992	75.67055	84.77775	75.5521	86.1823	96.10502	95.32002
σ	5.836306	6.541572	4.52804	3.809478	7.791801	4.813338	2.807494	3.966826

Sample	HeLa cell / TPE-PEGA-Hyd-DOX prodrug, Figure 7e							
Time	48 h							
C (mg/L)	100	10	1	0.1	0.01	0.001	0.0001	0
1	31.57895	58.33333	65	82.35294	83.33333	85.36585	81.57895	94.73684
2	10	58.33333	52.94118	75	80	80	87.5	92.5
3	45.83333	45.45455	54.54545	76.31579	86.2069	79.54545	85.71429	94.59459
4	30.76923	45.45455	50	75.75758	93.33333	86.04651	85.29412	92.68293
5	37.5	58.33333	81.48148	72.5	70.73171	85	80.64516	100
6	35.71429	58.33333	77.27273	78.04878	87.09677	88.63636	96.875	94.73684

7	45.83333	58.33333	78.94737	74.35897	86.11111	87.23404	91.42857	97.05882
8	28	41.66667	65.21739	77.14286	78.37838	82.35294	94.28571	94.44444
9	27.27273	58.33333	52.94118	71.79487	78.04878	80.85106	90.625	87.5
10	59.25926	58.33333	58.82353	74.28571	77.14286	82.97872	87.09677	94.11765
11	29.62963	30	72.22222	77.14286	86.11111	84.09091	85.29412	92.30769
12	48	11.11111	50	82.35294	78.78788	79.48718	92.59259	94.59459
μ	35.78256	48.50168	63.28271	76.42111	82.10685	83.46575	88.24419	94.1062
σ	12.73435	15.02206	11.76236	3.332005	6.039078	3.096509	4.98685	2.959176

Sample	HeLa cell / TPE-PEGA-Hyd-DOX prodrug, Figure 7e							
Time	72 h							
C (mg/L)	100	10	1	0.1	0.01	0.001	0.0001	0
1	15	44.23077	52.08333	68.57143	72.10526	85.36585	91.17647	97.05882
2	7.407407	31.42857	53.33333	62.5	85.12195	88.09524	100	92.30769
3	9.677419	23.68421	50.98039	70	88.57143	92.68293	93.33333	88.57143
4	16.12903	31.42857	61.70213	63.88889	82.2973	93.54839	100	94.28571
5	16.66667	24.24242	54.90196	66.66667	85.29412	91.17647	94.11765	92.30769
6	10.52632	27.27273	55.31915	76.31579	85.36842	89.13043	96.55172	91.42857
7	15.625	31.57895	58.69565	65.78947	88.57143	84.09091	90.625	97.05882
8	13.7931	31.70732	56.14035	64.86486	87.36842	92.10526	91.42857	94.59459
9	19.44444	34.09091	56.25	75.67568	84.09091	95.12195	94.11765	92.85714
10	18.51852	34.21053	54.71698	72.5	84.09091	91.17647	97.14286	85.29412
11	9.375	35.71429	58	63.63636	88.57143	88.09524	93.93939	97.36842
12	13.33333	39.02439	56.36364	71.42857	89.74359	89.74359	94.73684	97.4359
μ	13.79135	32.38447	55.70724	68.48648	85.0996	90.02773	94.76412	93.38074
σ	3.82471	5.821665	2.922172	4.71213	4.687761	3.270883	3.13885	3.773181

Sample	NIH3T3 cell / TPE-PEGA-Hyd-DOX prodrug, Figure 7f							
Time	24 h							
C (mg/L)	100	10	1	0.1	0.01	0.001	0.0001	0
1	73.52941	94.28571	94.59459	87.5	100	95.45455	92.85714	86.27451
2	77.08333	83.78378	83.78378	91.42857	93.33333	92.68293	96	88.09524
3	75.75758	79.41176	91.17647	100	94.44444	94.87179	97.91667	94.73684
4	86.66667	89.28571	94.87179	92.59259	90.47619	97.22222	100	92.68293
5	86.11111	90	92.10526	91.17647	92.5	83.72093	88.63636	97.22222
6	68.42105	91.17647	97.14286	90.2439	96.9697	88.63636	94.44444	89.3617
7	75.75758	80.76923	91.17647	90.47619	85.29412	92.68293	89.3617	97.36842
8	81.25	97.14286	96.9697	94.59459	89.74359	94.87179	91.48936	97.5
9	86.11111	88.88889	97.22222	92.5	84.09091	85.36585	93.54839	100
10	84.09091	75	85.29412	88.57143	100	94.11765	92.68293	95.45455
11	85.36585	95	92.30769	92.10526	88.57143	97.2973	95.34884	92.30769
12	77.19298	96	96.875	100	85.36585	93.93939	100	89.13043
μ	79.77813	88.39537	92.79333	92.59908	91.73246	92.57197	94.35715	93.34454
σ	5.984515	7.157816	4.508053	3.924898	5.481747	4.399202	3.713084	4.388587

Sample	NIH3T3 cell / TPE-PEGA-Hyd-DOX prodrug, Figure 7f							
Time	48 h							
C (mg/L)	100	10	1	0.1	0.01	0.001	0.0001	0
1	78.78788	89.47368	94.28571	92.10526	92.30769	92.5	97.22222	87.17949
2	75	89.3617	86.36364	94.73684	94.28571	93.10345	97.05882	93.93939
3	63.33333	84.375	96	84.21053	89.47368	94.28571	96.875	97.05882
4	84.84848	78.125	93.10345	82.22222	92.5	94.11765	94.87179	100
5	60	79.48718	93.93939	86.66667	94.87179	92.10526	100	92.10526
6	89.47368	77.77778	94.44444	83.87097	94.59459	82.75862	97.61905	92.15686
7	71.42857	84.61538	86.48649	93.93939	96.55172	95.45455	94.59459	93.93939
8	82.14286	87.5	88.46154	96.66667	100	87.09677	96.9697	97.05882
9	65	86.48649	89.74359	86.2069	83.87097	91.30435	94.28571	94.87179
10	76.47059	90	93.10345	92.10526	91.17647	100	100	100
11	77.77778	77.41935	77.41935	97.05882	94.59459	97.36842	96.875	97.82609
12	77.27273	89.47368	85.71429	92.30769	88.88889	94.44444	100	97.05882
μ	75.12799	84.50794	89.92211	90.17477	92.75968	92.87827	97.19766	95.26623
σ	8.844938	5.022027	5.338282	5.249474	4.132947	4.498375	2.01544	3.689568

Sample	NIH3T3 cell / TPE-PEGA-Hyd-DOX prodrug, Figure 7f							
Time	72 h							
C (mg/L)	100	10	1	0.1	0.01	0.001	0.0001	0
1	46.15385	90.47619	87.17949	92.68293	94.28571	94.59459	100	91.42857
2	87.09677	91.30435	92.5	87.5	90.32258	94.87179	97.36842	97.22222
3	63.63636	82.92683	88.37209	84	96.875	97.4359	88.63636	100
4	66.66667	84	89.74359	92.30769	88.88889	93.54839	88.88889	96.9697
5	54.54545	85.71429	90.32258	93.33333	92.68293	100	94.59459	97.22222
6	66.66667	87.5	92.30769	88.37209	97.36842	85.41667	96.66667	94.87179
7	70.83333	90.2439	92.10526	93.75	97.61905	97.36842	96.15385	100
8	73.68421	100	91.11111	92.85714	96.15385	92.10526	91.17647	97.36842
9	65.38462	79.54545	97.4359	96.42857	91.17647	97.5	100	100
10	67.56757	88.09524	90	86.11111	94.44444	100	97.2973	89.74359
11	59.09091	83.33333	96.66667	96.9697	97.36842	88.09524	96.875	97.2973
12	82.85714	85.71429	84.375	100	100	94.59459	94.44444	100
μ	67.0153	87.40449	91.00995	92.02605	94.76548	94.62757	95.17517	96.84365
σ	11.20633	5.287481	3.657558	4.7341	3.401821	4.436178	3.826671	3.360953
